# Molecular logic of the Zur-regulated zinc deprivation response in *Bacillus subtilis*

**DOI:** 10.1038/ncomms12612

**Published:** 2016-08-26

**Authors:** Jung-Ho Shin, John D. Helmann

**Affiliations:** 1 Department of Microbiology, Cornell University, Wing Hall, Ithaca, New York 14853-8101, USA

## Abstract

Bacteria respond dynamically to the changes in zinc availability. Repression by the *Bacillus subtilis* transcription factor Zur requires Zn(II), which binds with negative cooperativity to two regulatory sites per dimer to form, sequentially, Zur_2_:Zn_3_ and Zur_2_:Zn_4_ forms of the repressor. Here we show that, as cells transition from zinc sufficiency to deficiency, operons regulated by Zur are derepressed in three distinct waves. The first includes the alternative RpmEB(L31*) and RpmGC(L33*) ribosomal proteins, which mobilize zinc from the ribosome, whereas the second includes the ZnuACB uptake system and the YciC metallochaperone. Finally, as zinc levels decrease further, the Zur_2_:Zn_3_ form loses Zn(II) leading to derepression of RpsNB(S14*) and FolE2, which allow continued ribosome assembly and folate synthesis, respectively. We infer that zinc mobilization from intracellular zinc stores takes priority over energy-dependent import, and our results link the biochemistry of zinc sensing by Zur to the molecular logic of the zinc deprivation response.

## Abstract (web summary)

The transcription factor Zur controls the zinc deprivation response in *Bacillus subtilis*. Here, Shin and Helmann show that Zur-regulated genes are derepressed in three waves in response to zinc deprivation, and this is linked to the biochemistry of zinc sensing by Zur.

## Graphical abstract

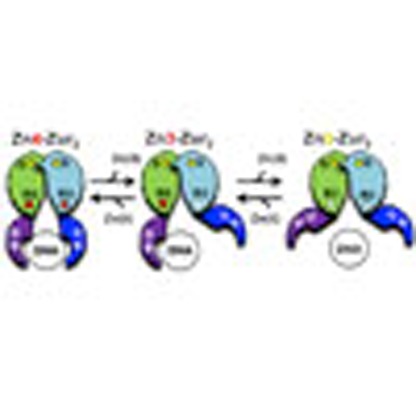

A hallmark of life is the ability to adapt to changing environmental conditions, often by altering the expression of DNA through the action of transcription factors. In many systems, we understand in atomic detail how activators and repressors are converted from their inactive to their active forms, and genomics-level approaches, such as transcriptomics and chromatin immunoprecipitation (ChIP), provide an overview of the complete suite of genes (regulon) that is affected by individual transcription factors. A quantitative understanding of transcriptional control mechanisms also requires an appreciation for the ways in which transcription factors are regulated (often involving integration of multiple inputs), the combinatorial nature of their interactions at specific regulatory regions and the graded responses resulting from variations in operator occupancy, as a function of transcription factor activity. Here we focus on a relatively simple system, the regulation of the zinc deficiency response mediated by the *Bacillus subtilis* zinc uptake regulator (Zur)^1^,^2^,^3^.

*B. subtilis* Zur is representative of a large class of bacterial metal-sensing transcription factors (metalloregulatory proteins) whose DNA-binding activity is regulated by the reversible binding of metal ions^4^. These proteins therefore function as ‘one-component' regulators that serve to directly couple changes in concentration of a simple ligand (an inorganic ion) to DNA occupancy^5^. *B. subtilis* Zur, like other members of the Fur family of metalloregulatory proteins, is a dimer in solution and requires a structural Zn(II) ion for protein folding and dimerization^3^. When Zn(II) levels are sufficient, Zn(II) is additionally bound to a regulatory site within each monomer and the resulting, fully metallated protein (designated Zur_2_:Zn_4_) is an active repressor. Zur is expressed at relatively uniform level across a range of growth conditions^6^, and its activity is regulated primarily, if not exclusively, by the reversible binding of Zn(II) ions to this regulatory binding site. As cells transition from Zn(II) sufficiency to deficiency, Zur transitions first to a partially metallated dimer (Zur_2_:Zn_3_) and finally to the inactive resting form with Zn(II) bound only at the structural sites, Zur_2_:Zn_2_. The presence of the Zur_2_:Zn_3_ intermediate results from the ∼20-fold negative cooperativity between the binding of the first and second Zn(II) ions to the regulatory sites in the Zur dimer^3^.

Zur is known to regulate at least seven operons encoding proteins that facilitate adaptation to Zn(II) limitation^2^,^7^,^8^,^9^. As might be anticipated for a stress response activated when an essential metal becomes limiting for growth, a key part of this adaptive response is the expression of a high-affinity ABC transporter uptake system encoded by the *znuACB* operon, which likely functions together with a Zn(II) scavenging lipoprotein, ZinT^10^,^11^,^12^, and a highly abundant member of the COG0523 family of metallochaperones (YciC) conserved in Bacteria, Eukarya and Archaea^13^. Adaptation to Zn(II) deficiency also commonly involves the expression of alternative ribosomal protein paralogues that functionally replace ribosomal proteins that require Zn(II) for function^14^,^15^. In *B. subtilis*, two Zur-regulated ribosomal proteins are paralogues of L31 (designated L31* and encoded by *rpmEB*) and L33 (designated L33*, encoded by *rpmGC*). Induction of L31* and L33* serves to displace the cognate Zn(II) containing proteins from the surface of the assembled ribosome thereby mobilizing Zn(II) into the cytoplasm for redistribution^16^,^17^. The expression of a third ribosomal protein paralogue (S14*, encoded by *rpsNB*) allows for *de novo* ribosome synthesis, which might otherwise become limited by the cell's ability to synthesize the Zn-requiring S14 protein^7^,^18^. Another process that becomes limiting when cells are zinc deficient is folate biosynthesis due to the Zn dependence of the FolE1 GTP cyclohydrolase. Zur also regulates an alternate, Zn-independent FolE2 enzyme^19^. In general, the replacement of enzymes dependent on one metal ion by an alternative enzyme dependent on a different metal or a non-metal cofactor is a widely conserved mechanism to facilitate adaptation to metal-limiting growth conditions^20^.

We previously speculated that the presence of negative cooperativity in Zn(II) binding might provide a mechanism for a graded response to changing Zn(II) levels^3^. Here we have tested this hypothesis and monitored the relative sensitivity of each of the Zur-regulated operons to zinc deprivation. Our results indicate that as cells transition from zinc sufficiency to zinc limitation operons regulated by Zur are derepressed in three distinct waves. First, induction of RpmEB(L31*) and RpmGC(L33*) mobilizes Zn(II) from the ribosome and ZinT primes the cell for Zn(II) import. Next, the high-affinity ZnuACB transporter for Zn(II) import is expressed together with the YciC metallochaperone. Finally, RpsNB(S14*) and FolE2 are expressed. Studies with strains expressing a mutant Zur protein (C84S) that can form the partially active Zur_2_:Zn_3_ form, but which is impaired in its ability to transition to the fully active Zur_2_:Zn_4_ form, indicate that this partially active form is still sufficient to repress the *rpsNB* and *folEB* genes *in vivo* and still binds with high affinity to the corresponding regulatory regions *in vitro*. We conclude that the negative cooperativity in Zn(II) binding to Zur is responsible for the transition between the middle and late genes derepressed as part of the zinc limitation adaptive response.

## Results

### The Zur regulon is derepressed in a stepwise manner

**Figure 1:**
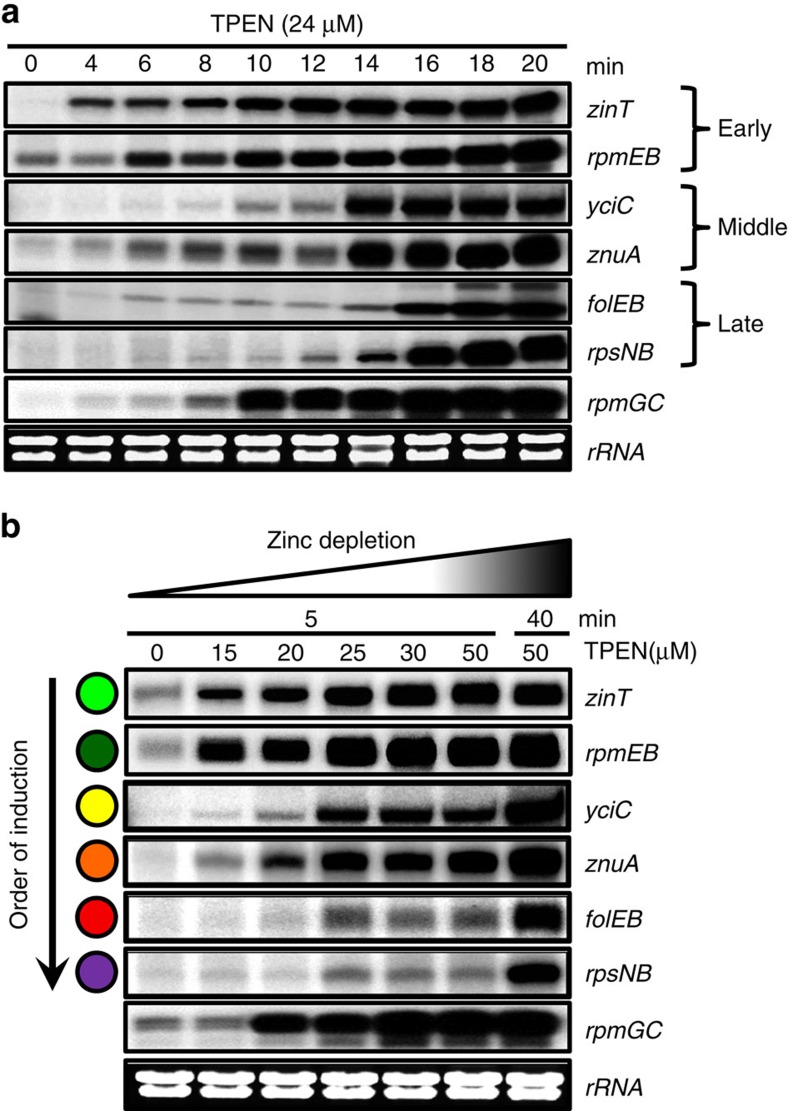
Ordered derepression of Zur target genes in response to Zn(II) deprivation. Wild-type cells were grown to early exponential phase (OD_600_ ∼0.5) in liquid LB medium and treated with the zinc chelator TPEN, before performing an S1 nuclease protection assay. (**a**) Cells were treated with 24 μM TPEN for 4–20 min (as indicated). (**b**) Cells were treated with variable levels of TPEN (15–50 μΜ) for 5 min or with 50 μΜ for 40 min to fully induce all Zur target genes. For each sample, RNA was isolated and analysed by S1 nuclease protection to quantify the expression level of each gene. Average values from three independent experiments are presented in Supplementary Figs 1 and 2 and representative full gel images are shown in Supplementary Figs 7 and 8.

We used an S1 nuclease protection assay to monitor the induction of six different Zur-regulated operons in cells exposed to the zinc chelator TPEN for various periods of time (Fig. 1a). The results reveal that Zur's target genes are induced in three waves which we assign as early (*zinT* and *rpmEB*), middle (*yciC* and *znuA*) and late genes (*folEB* and *rpsNB*) with respect to their time of induction after chelator addition. While these genes differ in their basal level of expression under zinc sufficient conditions (no added TPEN), they are all highly induced by TPEN but with different kinetics. On the basis of these findings, we extended this analysis to include an additional Zur-regulated gene, *rpmGC*, which is a pseudogene in *B. subtilis* 168 due to a frameshift mutation. Although *rpmGC* does not encode a functional protein in this genetic background, its expression is still regulated by Zur^7^. Since the *rpmEB* gene (encoding the L31* ribosomal protein) is induced early, we hypothesized that *rpmGC* (encoding L33*) would also be induced early. Indeed, *rpmGC* is induced at a time intermediate between the two early genes and the middle genes noted above (Fig. 1a). These results are supported by studies in which cells were treated for a fixed time (5 min) with increasing amounts of chelator (Fig. 1b). Average values from three independent experiments are presented in Supplementary Figs 1 and 2 and representative full gel images are shown in Supplementary Figs 7 and 8, which revealed a similar stepwise derepression.

### Stepwise derepression correlates with operator occupancy

**Figure 2:**
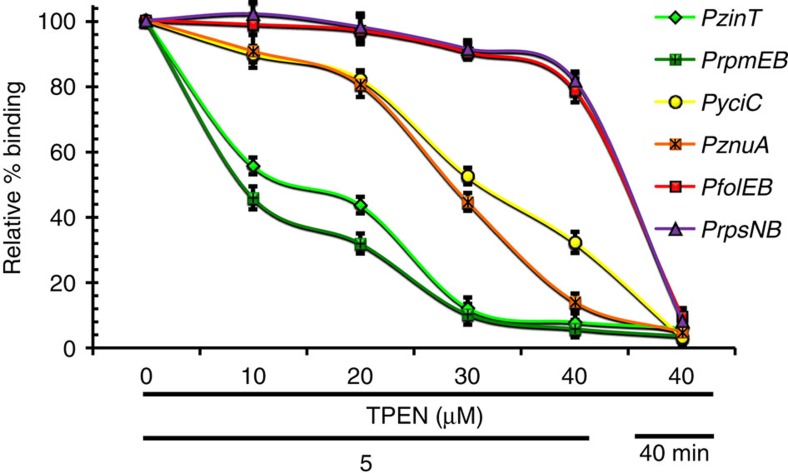
Operator occupancy correlates with *in vivo* expression. Zur occupancy on target promoters was assessed by *in vivo* crosslinking of cells followed by chromatin immunoprecipitation (ChIP) using anti-FLAG antibodies. Co-immunoprecipitated DNA was quantified using qPCR with primers to amplify the promoter regions of each target gene (Supplementary Table 2). Enriched DNA values for each Zur target gene from three independent experiments were normalized to the input (1% of the amount used for each ChIP experiment), and are presented as the relative per cent (%) of binding compared with the nontreated sample (full Zur binding; 100%).

The simplest model to account for the stepwise induction of the Zur regulon is that binding of Zur to its various operator sites is differentially affected as a function of zinc depletion. To monitor the occupancy of Zur protein on its operator sites *in vivo*, we used ChIP. As predicted, the operator occupancy at the six tested sites declined in parallel with the observed increase of mRNA expression level (Fig. 2). Indeed, the same three sets of early, middle and late genes can be discerned. These results are supportive of a model in which mRNA levels of Zur-regulated operons are modulated primarily, if not exclusively, by the relative occupancy of Zur at its operator sites.

### Role of negative cooperativity of Zn(II) binding to Zur

Previously, we demonstrated that Zur forms a dimer and that efficient dimerization requires a structural Zn(II) site^3^. The Zur_2_:Zn_2_ protein binds operator DNA with low affinity and mutations that prevent binding of Zn(II) to the regulatory site are non-functional in repression *in vivo*. The affinity of Zur for a cognate operator site (*folEB*) was shown to increase by 1,000-fold in the presence of Zn(II) sufficient to form the active Zur_2_:Zn_4_ form of the repressor^3^. Biochemical measurements revealed that the binding of Zn(II) to the regulatory sites (one in each monomer) occurs with negative cooperativity with a ∼20-fold difference in the measured affinities. Further, we found that a C84S mutation to one of the Zn(II) ligands of the regulatory metal binding site did not significantly affect binding of the first Zn(II), but greatly impaired the second binding event (a decrease in affinity of ∼10^4^-fold). Since the C84S mutant protein was still able to repress a Zur-regulated target (*yciC*) *in vivo*, we concluded that this protein likely retained some repressor activity despite being impaired in the transition from the Zur_2_:Zn_3_ to the Zur_2_:Zn_4_ form of the repressor^3^.

**Figure 3:**
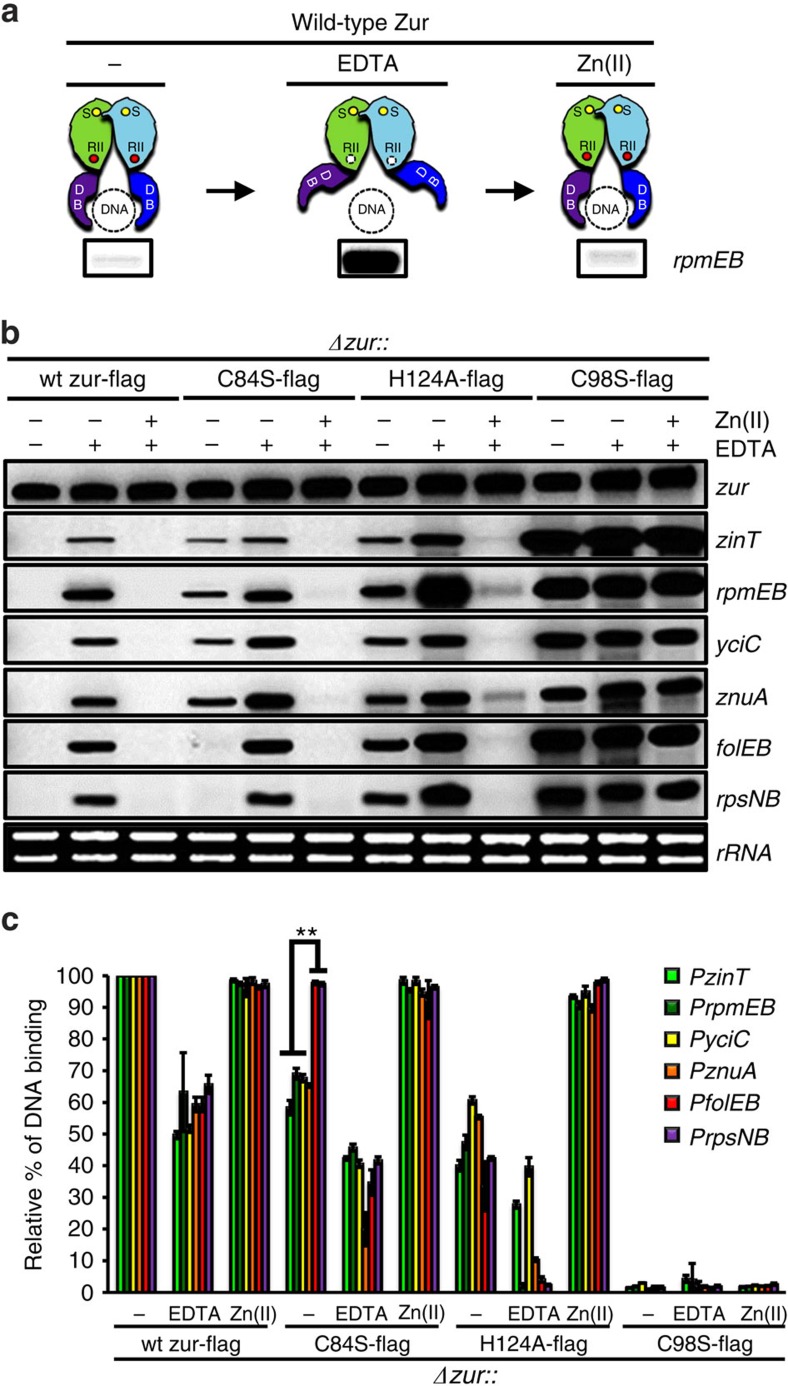
Comparative repressor activity of mutant Zur proteins. (**a**,**b**) We compared the ability of FLAG-tagged variants of the wild-type (WT) Zur and the C84S mutant to repress each of the six Zur-regulated operons using S1 nuclease protection. (**a**) In WT, the general expression pattern of *rpmEB* in LB liquid medium (repressed), after treatment with 2 mM EDTA (derepressed), and then subsequently resuspended in LB medium amended with 25 μM ZnSO_4_ (repressed). (**b**) Expression profiles of six Zur target genes in complemented strains with FLAG-tagged WT or mutant Zur proteins under the same conditions as (**a**). Average values from three independent experiments are presented in Supplementary Fig. 3a–c. Transcripts of the *zur* gene and ribosomal RNA were used as constitutive expression controls. (**c**) To monitor the *in vivo* DNA-binding activity of FLAG-tagged Zur variants, cells were grown under the same condition as **a** and **b**, and were analysed by *in vivo* crosslinking and immunoprecipitation as described in Fig. 2. The normalized values from three independent experiments were determined relative to input (1%) and are presented as per cent (%) of DNA binding relative to the untreated control cells.

One possible mechanism to enable stepwise repression of Zur-regulated genes would be differential activity of the Zur_2_:Zn_3_ and Zur_2_:Zn_4_ forms of the repressor. To explore this possibility, we compared the ability of FLAG-tagged variants of the wild-type (WT) Zur and the C84S mutant to repress each of the six Zur-regulated operons using S1 nuclease protection (Fig. 3; full gel images in Supplementary Fig. 9). As controls, we also included Zur mutants shown previously to be defective for binding the structural Zn(II) ion (C98S) or compromised for protein dimerization (H124A). To monitor the basal level of repressor activity in standard growth conditions, cells were grown to mid-logarithmic phase (OD_600_=0.5) in Luria-Bertani (LB) medium. Zinc limitation was imposed by the treatment with 2 mM EDTA for 1 h, and washed cells were then resuspended in LB medium amended with 25 μM Zn(II) for 1 h to restore repression (Fig. 3a). Unlike TPEN, EDTA does not enter cells and imposes zinc limitation by chelating extracellular zinc. Under these conditions, WT Zur was able to repress all six operons with the ambient zinc levels in our LB medium (∼18 μM), consistent with the previous work^1^,^2^,^7^. All six tested operons were induced by EDTA, leading to mRNA accumulation, and this was reversed by resuspension in zinc-amended medium (Fig. 3b, lanes 1–3). In contrast, in cells expressing the C84S Zur mutant both the early and middle genes were partially derepressed in LB medium, whereas the late genes (*folEB* and *rpsNB*) were fully repressed. Again, all six operons were derepressed by EDTA and repression was complete in the zinc-amended medium (lanes 4–6). These results suggest that the C84S mutant, which is likely to be largely restricted to the Zur_2_:Zn_3_ form in unamended medium, is still active in repression of the late genes, but not the early and middle genes. In contrast to the C84S mutant, a Zur protein defective for dimerization (H124A), is reduced in activity at all six genes (lanes 7–9), and a mutant defective for coordinating the structural zinc ion (C98S) is grossly defective in function (lanes 10–12), despite similar levels of expression of all Zur proteins under all tested conditions (Supplementary Fig. 3e).

To confirm that the effects observed at the level of mRNA are reflective of Zur operator occupancy, we conducted ChIP experiments using the four FLAG-tagged Zur variants (Fig. 3c). The fractional enrichment of each operator site (relative to that observed for WT Zur in LB medium) is consistent with the observed mRNA levels: EDTA treatment leads to a substantial (but not full) decrease in operator occupancy (approximately twofold) for both WT and C84S, consistent with the observed derepression. Further, the difference in repression efficiency in LB medium noted for C84S Zur (Fig. 3b) was also reflected in the promoter occupancy levels (Fig. 3c, lane 4). As expected, the H124A Zur (dimerization defective) had decreased operator occupancy under most conditions (but could be rescued by excess zinc), and the C98S Zur (disrupted in the structural Zn-binding site essential for protein folding and dimerization) was essentially inactive in this operator binding in this assay.

### Stepwise Zn(II) binding and operator discrimination

The above results are consistent with the hypothesis that decreasing zinc availability leads to a decrease in fully metallated Zur (Zur_2_:Zn_4_) and that this leads to a selective induction of early and middle genes, whereas the remaining Zur_2_:Zn_3_ population may suffice for repression of late genes. To further explore the basis for this graded response, we sought to determine the biochemical affinity of Zur proteins for each operator. All four Zur proteins (WT, C84S, C98S and H124A) were purified after overproduction in *Escherichia coli* and biochemically characterized using assays to monitor purity, dimerization and Zn content as described previously for Zur and other Fur family proteins^3^,^21^,^22^ (Supplementary Fig. 4). Next, the DNA-binding affinity was monitored for each active protein (the C98S protein was inactive in binding DNA) at the six operator sites using an electrophoretic mobility shift assay (EMSA) optimized with WT Zur (Supplementary Figs 5 and 6). The results, summarized in Table 1, indicate that WT Zur bound to all six operator sites with similar affinities (*K*_d_ values of ∼12–25 nM) that differed by no more than approximately twofold. The H124A Zur protein, which was very poorly active as a repressor *in vivo* (Fig. 2), bound with much reduced affinity (*K*_d_ values ∼100 nM). Of note, the C84S mutant protein was essentially unaffected in its ability to bind to the operators of the two late genes, *folEB* and *rpsNB*, but had reduced affinity for the other four operator sites. These findings support the inference that declining cellular zinc levels lead to an initial transition of Zur_2_:Zn_4_ to Zur_2_:Zn_3_, and the residual activity of the Zur_2_:Zn_3_ form can account for the comparatively slow induction of late genes. However, the operator affinities in this assay do not provide an explanation for the difference in sensitivity between early and middle genes. We note that early and middle genes clearly differ in their operator occupancy as a function of zinc depletion *in vivo* (Fig. 2), yet do not detect any difference in operator affinity *in vitro* that can account for these differences, at least under fully zinc sufficient conditions (Table 1).

### Stepwise repression is independent of local chromosomal context

**Figure 4:**
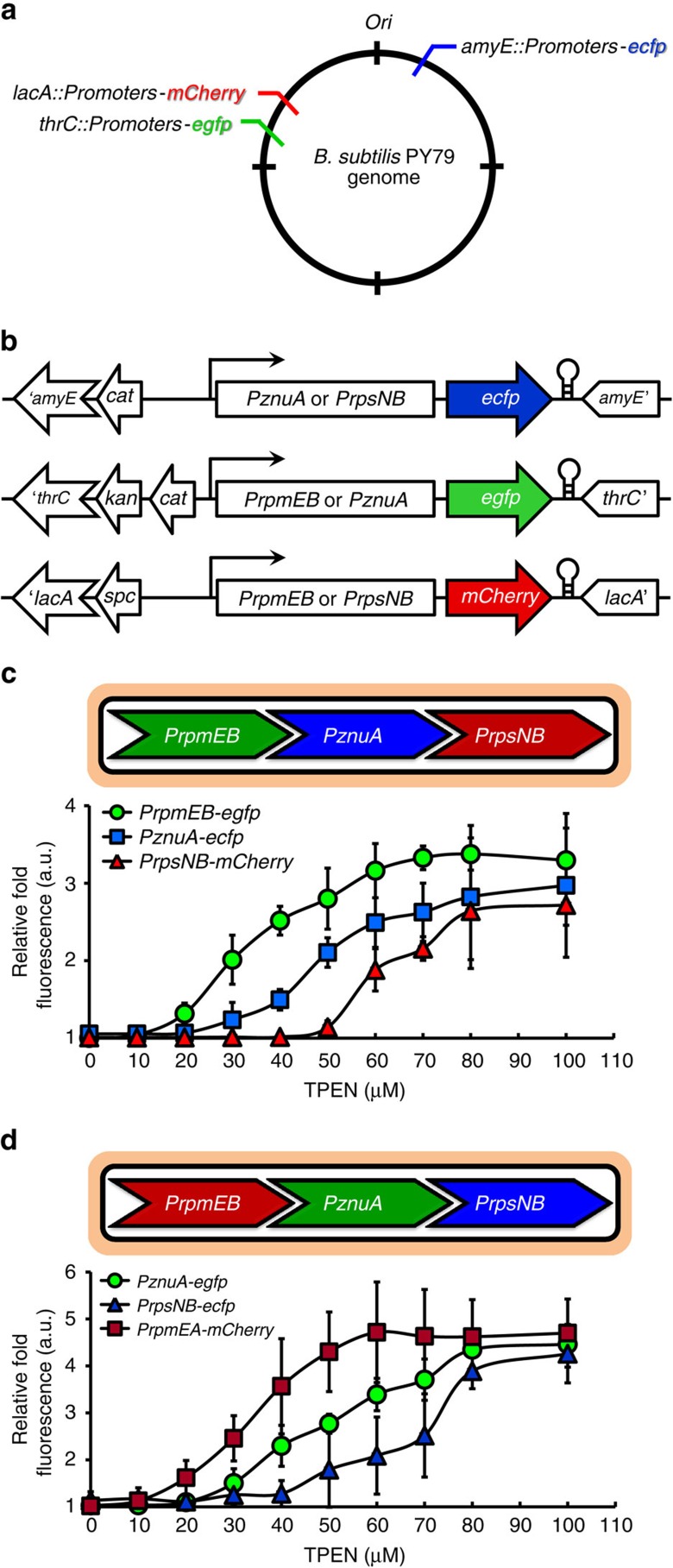
Confirmation of stepwise gene regulation using fluorescent reporter fusions. (**a**) Genomic loci for distinct integration and ensemble of differential fluorescence proteins in *B. subtilis* PY79. (**b**) Promoter regions of representative early, middle and late genes were used to express each indicated fluorescent protein using primer pairs in Supplementary Table 2. All promoter fusions were integrated via double crossover events at non-essential loci (*amyE*, *thrC* or *lacA*). The flanking regions of *amyE*, *thrC* or *lacA* were oriented in the opposite direction of the promoter fusion proteins to avoid spurious transcriptional signals, and a transcriptional terminator from phage lambda was inserted downstream of the flanking genes. (**c**,**d**) Relative average fold fluorescence (a.u.) from three independent experiments of each promoter fusion are presented after subtraction of background as determined with promoter-less vector control strains.

Although our working model is that the temporal induction of genes in the Zur regulon is due exclusively to binding of Zur to the promoter proximal operator site(s), we cannot exclude the possibility that other factors might influence their regulation and, specifically, their sensitivity of zinc depletion. For example, it is increasingly appreciated that chromosome position can affect gene function in bacteria^23^, and regulators and nucleoid-associated proteins can function over extended distances to impact regulation. To determine whether the local sequence context of each operon is sufficient to determine its sensitivity to zinc-mediated repression, we generated transcriptional fusions of representative early, middle and late promoters to fluorescent protein reporters and integrated these at three distinct sites around the chromosome. In both strains tested, the reporter fusions retained their relative sensitivity to zinc depletion (Fig. 4). This observation indicates that chromosome context is not determinative, and further reveals that all information needed for the stepwise induction of the Zur regulon is contained within the ∼500 bp regions used for fusion construction.

## Discussion

**Figure 5:**
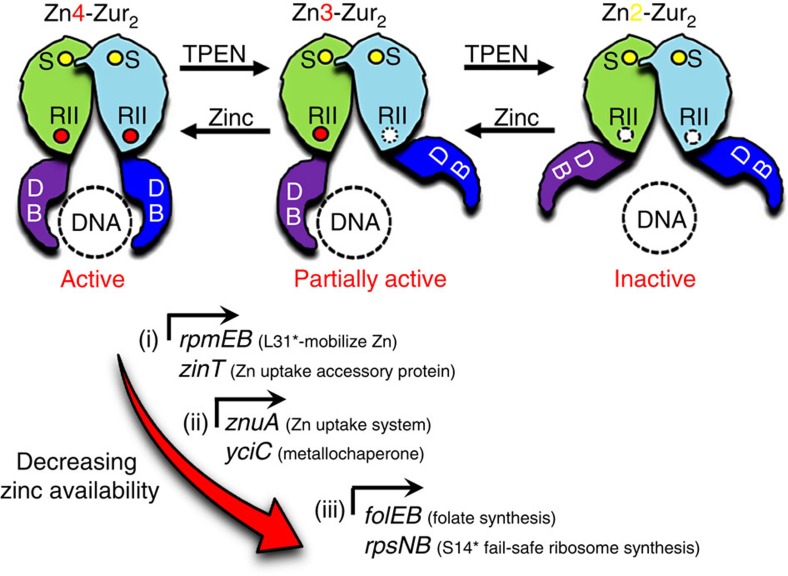
Proposed stepwise regulation of Zur target genes. Zur_2_:Zn_4_ indicates the fully active Zur dimer, containing four Zn(II) ions per dimer in both structural (S) and regulatory Zn(II) site (RII). Zur_2_:Zn_4_ represses all Zur target genes. Zur_2_:Zn_3_ is the partially active form of Zur dimer after the loss of one Zn(II) ion from the regulatory Zn(II) site in one protomer in the dimer. Zur_2_:Zn_2_ represents the inactive form that results from the loss of both regulatory Zn(II) ions per Zur dimer resulting in transcriptional derepression *in vitro* and *in vivo*.

The observation that Zur-regulated genes are derepressed in a distinct temporal order as cells transition from sufficiency to deficiency provides new insights into the molecular logic of the zinc deprivation response (Fig. 5). Our results indicate that as intracellular zinc availability declines the first response is the mobilization of zinc from surface-associated, zinc-associated ribosomal proteins. Both L31 and L33 have been previously implicated as part of a widely distributed but underappreciated mechanism of zinc storage^7^,^14^,^16^,^17^,^24^. Just as cells may store iron in times of sufficiency within ferritin and ferritin-like proteins, many bacteria appear to use small zinc-binding proteins that associate with the surface of the ribosome as a mobilizable Zn(II) store^15^. These storage proteins are notable since they are small peptides (<50 amino acids in length) that coordinate Zn(II) through two Cys–X–X–Cys motifs. In many organisms, there are paralogues of nearly identical sequence that lack many or all of these cysteine ligands and these non-Zn(II) binding paralogues are nearly always regulated by Zur or analogous sensors of zinc deprivation^15^. Biochemical studies reveal that these paralogous ribosomal proteins (for example, L31*) can displace the endogenous ribosomal protein from the surface of the ribosome to mobilize the associated Zn(II)^16^. Genetic and physiological studies support a model in which this mobilized Zn(II) is important for growth under conditions of zinc limitation^7^. It is presently unknown whether Zn(II) exchange from the displaced L31 is spontaneous or whether it is facilitated by specific low-molecular-weight ligands or protein chaperones or by proteolysis. Regardless of mechanism, due to the high abundance of ribosomes in bacterial cells (∼2–6 × 10^4^ per cell depending on growth rate), it can be calculated that ribosomally associated Zn(II) represents a substantial fraction of total cell-associated Zn(II). The other protein induced as part of the early response to Zn(II) deprivation is ZinT. ZinT is a surface-associated lipoprotein hypothesized to function as an accessory factor for Zn(II) import^10^,^11^,^12^. It is somewhat surprising that ZinT would be induced before the ZnuACB uptake system with which it presumably functions. It may be that ZinT primes the cell for Zn(II) import by scavenging Zn(II), or ZinT may function with other transporters.

As cellular zinc levels decline further, and Zn(II) mobilized from the ribosomal pool is consumed by continued protein synthesis, the middle genes are induced. These include the high-affinity Zn(II) ABC transporter, ZnuACB, and the putative metallochaperone YciC. The function of YciC is presently unclear, but this protein is representative of a highly conserved family of GTPases implicated as cofactors for metal insertion into target metalloproteins^13^. YciC may be involved in the intracellular trafficking of Zn(II), which likely becomes more critical when zinc is limiting for growth. One possible role of YciC is to prioritize Zn(II) utilization by making sure that Zn(II) is delivered to those enzymes essential for growth. An analogous problem arises with respect to iron deprivation. In this case, *B. subtilis* activates an iron-sparing response involving the small RNA FsrA and accessory proteins FbpABC^25^. This system translationally represses numerous abundant iron-containing enzymes and complexes including succinate dehydrogenase, glutamate synthase and lactate dehydrogenase with the presumed function of enabling the limiting amounts of iron to be available for incorporation to more essential enzymes. An analogous iron-sparing response is present in *E. coli* mediated by the RyhB sRNA^26^,^27^. There is, to date, no evidence for an analogous zinc-sparing response in *B. subtilis*, and we speculate that one role of YciC may be to help the cell prioritize the allocation of limited zinc. However, zinc-sparing responses are present in other organisms: one well-documented example is in the model eukaryote *Saccharomyces cerevisiae* where abundant Zn(II)-containing alcohol dehydrogenases are downregulated in response to zinc limitation^28^.

The ability of the ZnuACB system to overcome Zn(II) limitation relies, of course, on the availability of extracellular zinc. As zinc levels fall further, the final two genes are induced, *rpsNB* and *folEB*. Repression of these genes is maintained at intermediate levels of intracellular Zn(II) by their unique ability to be efficiently repressed *in vivo* by the Zur_2_:Zn_3_ form of Zur. The *rpsNB* gene encodes an S14 paralogue (S14*). Unlike the L31 and L33 proteins, which are dispensable, S14 is essential and is required at an early stage of ribosome biogenesis. Thus, if cellular Zn(II) levels decline to levels that no longer support Zn(II) acquisition by S14 cells will be unable to assemble new ribosomes. The induction of *rpsNB* allows the synthesis of a replacement, Zn-independent S14* protein that enables new ribosome synthesis (a ‘failsafe' pathway)^18^. Mutant strains lacking *rpsNB* can still grow under severe zinc limitation, but their inability to synthesize new ribosomes results in linear rather than exponential growth^7^, consistent with the prediction that each new daughter cell inherits only half the ribosome complement of its parent. Like *rpsNB*, *folEB* encodes a replacement function for a protein that fails when zinc levels are low. The FolE1 enzyme (product of *folEA*) encodes a Zn(II)-dependent GTP cyclohydrolase required for folate biosynthesis. The *folEB* product is a non-orthologous replacement that allows continued folate synthesis even under conditions of severe zinc depletion^19^.

In summary, our results indicate the transition from zinc sufficiency to zinc deprivation derepresses (i) early proteins for the mobilization Zn(II) from the surface of the ribosome (L31* and L33*) and to prime the cell for zinc uptake (ZinT), (ii) middle proteins for high-affinity zinc import (ZnuACB) and an accompanying metallochaperone (YciC), and (iii) late proteins to replace critical functions that fail as zinc levels decline further, RpsNB and FolE2. Numerous other sites in *B. subtilis* are thought to be associated with Zur *in vivo*, as judged by ChIP, but the significance of this extended regulon is not clear, and many sites did not appear to significantly affect gene expression^9^. A graded response to zinc deprivation is also likely to occur in other bacteria. Indeed, a graded response of Zur has been previously documented in *Streptomyces coelicolor*^29^, although the molecular basis for this response, and its physiological implications, were not clear. Similarly, the *E. coli* Zur regulon contains operator sites of widely varying affinities and this has been correlated with the overall magnitude of the derepression response but not yet with a defined temporal order of induction^30^.

It is likely that graded responses are controlled by a combination of factors that ensure that genes are derepressed in an optimal order. Here we have documented the role of negative cooperativity in zinc binding to the two regulatory sites in each dimer in helping distinguish early and middle genes from late genes. However, our results have not yet provided a mechanism to explain the transition from early to middle genes, although this transition is also correlated with Zur operator occupancy (Fig. 2). In the case of some other Fur homologues (including *B. subtilis* Fur^31^, *S. coelicolor* Zur^29^ and *Magnetospirillum gryphiswaldense* Fur^32^) a second regulatory site within each monomer (site 3) may serve to fine-tune operator binding, with some sites requiring occupancy of this additional site and others not. An added level of complexity results from cooperativity in binding of multiple dimers to a single regulatory region. Although Fur proteins were originally proposed to bind to a 19 bp Fur box recognition sequence^33^, subsequent studies demonstrated that each a minimal Fur-binding site is a 7–1–7 inverted repeat and the classic 19 bp Fur box represents two overlapping repeats that can bind a dimer of dimers^34^. Other Fur family members likely share a similar architecture, at least at some operators^4^. Recent structural studies have highlighted the role of electrostatic interactions between the two dimers in cooperative binding by *E. coli* Zur^30^ and *M. gryphiswaldense* Fur^32^. Thus, another possible mechanism to impart a preferred order for derepression is a variable number of dimers or a variable level of cooperativity between dimers at a particular operator region. Indeed, in the *B. subtilis* Fur regulon, some operons are regulated by a single 7–1–7 repeat and bind a single Fur dimer, whereas other operons, such as *dhb*, are tightly repressed by a dimer of dimers^34^,^35^.

The idea of a graded response occurring as a function of increasing levels of a functional transcription factor is well precedented, but in only a minority of cases is the underlying logic apparent. One notable example is the regulation of gene expression in response to declining nutrient availability by the Spo0A transcription factor in *B. subtilis*. This is a complex system, involving multiple kinases, phosphotransfer proteins and phosphatases that integrates a variety of stress signals. As the level of the active Spo0A∼P transcription factor increases, cells are proposed to sequentially activate a motility response (sliding), biofilm formation and ultimately they enter into sporulation^36^. In this and related systems, a remaining challenge is to understand the molecular basis of this sequential gene regulation that may involve different forms of the transcription factor and variable affinities of activated factor for its operator sites, combinatorial effects with other regulators and complex promoter architectures.

## Methods

### Bacterial strains and culture conditions

All *B. subtilis* strains used in this study were isogenic with common laboratory strains listed in Supplementary Table 1. *B. subtilis* CU1065 was grown on LB medium and modified glucose minimal medium (20 g l^−1^ (NH_4_)_2_SO_4_, 183 g l^−1^ K_2_HPO_4_*3H_2_O, 60 g l^−1^ KH_2_PO_4_, 2 g l^−1^ MgSO_4_*7H_2_O, 10 g l^−1^ sodium citrate, 0.5% glucose, 0.5 mM CaCl_2_ and 5 μM MnCl_2_) was used for PY79 at 37 °C. When appropriate, antibiotics were included at: 100 μg ml^−1^ spectinomycin, 5 μg ml^−1^ chloramphenicol, 10 μg ml^−1^ kanamycin, 5 μg ml^−1^ tetracyclin and 1 μg ml^−1^ erythromycin plus 25 μg ml^−1^ lincomycin for the selection of various *B. subtilis* strains. DNA was transformed into *B. subtilis* using a modified version of a previously published protocol^37^. *E. coli* DH5α was used for routine DNA cloning^38^. Unless indicated otherwise, liquid media were inoculated from an overnight pre-culture and incubated at 37 °C with shaking at 200 r.p.m.

### Preparation of total RNA

Total RNA was isolated from *B. subtilis* strains that were cultured to mid-logarithmic phase (at an OD_600_ of 0.4–0.5) in LB medium. For Zn(II)-depleted condition, various amounts of TPEN were treated for 5 or 40 min. Total RNA was extracted by the ‘hot phenol method' as described^39^. The total amount of RNA and its quality were measured by absorbance spectroscopy and confirmed by resolving RNA samples on 1.3% formaldehyde agarose gels.

### S1 nuclease mapping analysis

Gene-specific DNA oligonucleotide probes for *zur*, *zinT*, *znuA*, *yciC*, *folEB*, *rpsNB* and *rpmEB* transcripts were used for PCR amplification using *B. subtilis* wild-type genomic DNA as template. The appropriate primer pairs are listed in Supplementary Table 2. An amount of 100 μg of total RNA was pelleted and lyophilized. Each specific DNA probe was radiolabelled with (γ-^32^P) ATP and T4 polynucleotide kinase, and 30,000–40,000 c.p.m. of labelled probe was used in each reaction. The total RNA pellet was carefully resuspended in 20 μl hybridization buffer (40 mM PIPES (pH 6.4), 400 mM NaCl, 1 mM EDTA, 80% (v/v) formamide). Individual samples were incubated at 95 °C for 25 min and slow cooled to 42 °C. Following incubation overnight, 300 μl of S1 nuclease mix containing 100 units of S1 nuclease in S1 nuclease buffer (280 mM NaCl, 30 mM NaOAc (pH 4.4), 4.5 mM ZnOAc) was added and incubated at 37 °C for 45 min. The reaction was terminated by addition of 75 μl of S1 nuclease termination solution (2.5 M NH_4_OAc, 0.05 M EDTA). The DNA–RNA hybrid was precipitated by adding 400 μl of isopropanol and the pellet was washed with 70% (v/v) ethanol, vacuum dried and resuspended in 13 μl alkaline loading dye. The protected DNA fragments were then resolved by 6% (wt/vol) polyacrylamide gels containing 7 M urea. The dried gels were exposed to a phosphor imaging screen (Typhoon FLA 7000; GE) and bands were quantified using Multi Gauge V3.0 (Fuji).

### Quantitative *in vivo* crosslinking and immunoprecipitation

*In vivo* crosslinking of DNA to Zur proteins and subsequent immunoprecipitation with specific antibody to FLAG were carried out. Cells were grown in LB medium to mid-logarithmic phase (at an OD_600_ of 0.5–0.6) and each 30 ml aliquots were spun down and the pellets were saved at −80 °C. For crosslinking, pellets were resuspended in buffer CA (10 mM Na_2_HPO_4_, 2 mM KH_2_PO_4_, 137 mM NaCl and 2.7 mM KCl (pH 7.4)) and 30 μl of 34% formaldehyde was added and the samples were gently rocked at room temperature for 10 min. To quench the crosslinking, 133 μl of 1 M glycine pH 7.5 (final molar concentration 0.133 M) was added, and the cells were shaken gently at 4 °C for 30 min. Cells were collected by centrifugation, and the pellets were resuspended and washed twice with buffer CB (50 mM Tris–HCl (pH7.4), 150 mM NaCl and 1 mM EDTA). Washed cells were resuspended in 0.5 ml buffer CB and lysed by sonication. Total 400 μl of supernatant was collected after centrifugation and frozen at −80 °C after aliquot. A measure of 1 μl of the lysate was diluted with 9 μl of the dilution buffer and the mixture was saved at −80 °C, to serve as the input-control (1% of input DNA). For immunoprecipitation, α-FLAG M2 magnetic agarose beads (M8823-Sigma) were washed and resuspended in 400 μl buffer CB. A measure of 200 μl cell lysate (total 200 μg of proteins) was mixed and incubated at 4 °C for overnight on rotation mixer. The bead slurry was recovered by using the magnetic stand for 1 min standing and washed twice with 400 μl buffer CB. The Zur–DNA complexes were eluted from the beads by addition of 0.1 M glycine–HCl (pH 3.0) and neutralized by buffer CC (500 mM Tris–HCl (pH 8.0) and 1.5 M NaCl). The input-control samples were also diluted with the elution buffer to the same final volume as the samples. Enriched target DNAs were purified using PCR Clean-Up Kits (Thermo Fisher Scientific) as per the manufacturer's instructions. Each 1 μl volume of eluted DNAs was quantified using the qPCR kit (Bio-Rad) followed by the manufacturer's instructions with primer pairs of *zur*, *zinT*, *znuA*, *yciC*, *folEB*, *rpsNB*, *rpmEB* and 16S rRNA in Supplementary Table 2. The data were normalized to values from 1% input.

### Resuspension experiments

BsZur variants with a C-terminal FLAG-tag were integrated at the *amyE* locus of CU1065 *zur::tet* (1). The strains were grown in LB medium to mid-logarithmic phase (at an OD_600_ 0.5) with appropriate antibiotics. Each 40 ml from cell culture was spoon down and saved before and after 2 mM EDTA treatment for 1 h for the nontreated control sample or Zn(II)-depleted cells. Remained cell culture was collected and the pellet was washed three times with equal volume of chelated LB medium. Resuspended cells with an equal volume of pre-warmed fresh LB medium containing 25 μM ZnSO_4_ were further incubated for another 1 h before harvesting.

### Structure modelling

Modelled three-dimensional structure of BsuZur dimer was constructed after sequence alignment of the structure of the closely related Zur protein of *S. coelicolor* (PDB ID 3MWM)^29^ and built using PROMOD-II and MODELLER (http://swissmodel.expasy.org). The final 3D structure model was represented in the cartoon using PyMOL viewer program (2008. Open free version of DeLano Scientific).

### Purification of BsZur wild-type and mutant proteins

Wild-type, C84S, C95S and H124A mutant Zur proteins were purified from *E. coli* BL21 (DE3) cells containing pET3a-based recombinant plasmid. The coding sequence for WT BsZur was cloned into pET3a (Novagen) between the NdeI and BamHI restriction sites. For BsZur mutants, plasmids were constructed using quick change site-directed mutagenesis and transformed into *E. coli* strain BL21 (DE3/pLysS). For the purification of Zur, an overnight culture from a single colony was used to inoculate 1 liter of LB medium. Cells were grown with vigorous shaking at 37 °C to an optical density at 600 nm (OD_600_) of 0.5 and were induced with 1 mM isopropyl-β-D-thiogalactopyranoside with 25 μM ZnSO_4_ for 6 h at 30 °C. Collected cells were resuspended with binding buffer (20 mM Tris–HCl (pH 7.9), 0.5 M NaCl, 1 mM TCEP {tris(2-carboxyethyl)-phosphine} and 5 mM imidazole) and cell extracts were prepared by sonication and ultra-centrifugation at 20,000 g for 30 min. Cell extracts were loaded onto a nickel-charged NTA column and then washed with six volumes of binding buffer followed by six volumes of washing buffer (20 mM Tris–HCl (pH 7.9), 0.5 M NaCl and 10 mM imidazole). Zur was eluted with 10 volumes of elution buffer (20 mM Tris–HCl (pH 7.9) and 0.5 M NaCl) containing linear imidazole gradients from 20 to 500 mM. Fractions containing Zur proteins were pooled and dialySed against buffer A (20 mM Tris–HCl (pH 7.8), 250 mM NaCl, 5% (vol/vol) glycerol and 4 mM EDTA)) to remove imidazole and nickel. For holo forms of Zur proteins, dialysed proteins in buffer A were further dialysed against buffer B (20 mM Tris–HCl (pH 7.8), 100 mM NaCl, 10% glycerol and 0.2 mM dithiothreitol (DTT)) and buffer C (20 mM Tris–HCl (pH 7.8), 50 mM NaCl, 30% glycerol, 1 mM DTT and 25 μM ZnSO_4_). The purified Zur proteins were concentrated by centrifugal filter devices (Millipore, 3,000 MW CO) before injection onto High load TM (16/60) pg Superdex G75 column in FPLC system (Pharmacia). The column was equilibrated with buffer G (20 mM Tris–HCl (pH 7.8), 50 mM NaCl, 2 mM DTT and 0.1 μM ZnSO_4_) so that the final buffer consisted of EMSA. Eluted fractions were monitored through ultraviolet detector. Concentrations of purified wild-type Zur and variant proteins were estimated in triplicates by Bradford assay (Bio-Rad) using bovine serum albumin (Sigma-Aldrich) as the calibration standard at A595. Measurement of ultraviolet absorbance at 280 nm, combined with calculated molar extinction coefficient (ɛ_280_=13,785 M^−1^ cm^−1^; http://expasy.org/cgi-bin/protparam), gave nearly identical values. The purity of each protein was confirmed through Coomassie blue staining of loaded protein samples in the SDS–polyacrylamide gel electrophoresis (SDS–PAGE) gel. The protein was stored in final storage buffer S (20 mM Tris–HCl (pH 7.8), 50 mM NaCl, 2 mM DTT, 30% glycerol and 0.1 μM ZnSO_4_) at −80 °C. To avoid metal contamination, all buffers were prepared through chelex-100 column (Bio-Rad).

### Gel-based dimerization assay

To confirm oligomeric forms of purified wild-type and mutant Zur proteins in solution^3^, total 7 μg of purified Zur proteins were loaded on the native non-reducing gel and visualized by Coomassie blue staining method. To keep multimeric forms of protein, non-boiled protein samples were loaded after mixed with non-reducing loading buffer (no DTT or β-mer). For fully reduced and denatured proteins, reducing buffer containing 140 mM β-mer and 2% SDS was added and then boiled at 95 °C for 10 min. For experimental control, reduced and fully denatured protein samples were loaded on to 13% SDS–PAGE after being mixed with the same amount of non-boiled proteins^40^.

### Detection of Zn(II) by PAR staining assay

To check Zn(II) occupancy in multimeric forms of Zur protein, resolved non-reducing SDS–PAGE gel was soaked in buffer PZ (20 mM Tris–HCl (pH 7.8), 50 mM NaCl and 5% glycerol) containing final 10 mM 4-(2-pyridylazo resorcinol (PAR)) for 20 min after washing with Milli-Q water two times. To release of Zn(II) ions from coordinating ligands, final 50 mM H_2_O_2_ was added and incubated for 20 min. PAR-stained images were taken on the white light every minute since the addition of H_2_O_2_.

### Electrophoretic mobility shift assays

Each Zur target promoter DNA probes of ∼45 bp containing Zur-binding sites were isolated using crush and soaking method from the polyacrylamide gel^41^ after annealing with each primer pairs in Supplementary Table 2. The purified DNAs were labelled at 5′-ends with (γ-^32^P) ATP using T4 polynucleotide kinase. Binding reactions were performed with ∼1 fmol of labelled DNA fragments and 0.075–616 nM of purified Zur proteins in 20 μl of the reaction buffer (20 mM Tris–HCl (pH 6.4), 50 mM KCl, 1 mM DTT, 0.1 mg of bovine serum albumin per ml, 5% glycerol and 0.1 μg of poly(dI-dC), with 0.1 μM ZnSO_4_). Following incubation at room temperature for 20 min, the binding mixture was subjected to electrophoresis at 4 °C on a 5% polyacrylamide gel in TA (pH 6.4) buffer. After electrophoresis, the dried gels were exposed and quantified by a phosphor image analyser (Typhoon FLA 7000). A band intensity of unbound DNA probes was measured against Zur concentration using Multi Gauge V3.0 software. Digitalized data were fit to binding curves through SigmaPlot 2001 program (SPSS Inc.). Apparent *K*_d_ values, corresponding to the concentration of variables (Zur) at half-maximal upshift of DNA probes, were determined from at least three independent sets of experiments.

### Construction of fluorescence fusion strains

For construction of reporter fusions between the representative Zur target promoters corresponding to groups with differential Zn(II) sensitivity and the three different fluorescent protein encoding genes (*ecfp*, *egfp* and *mCherry*), each promoter region was amplified by PCR using DNA oligonucleotides shown in Supplementary Table 2. To integrate at different loci, the PCR products were digested with XmaI restriction enzyme and ligated into three different integration vectors digested by same restriction enzyme. Constructs were confirmed by Sanger sequencing and transformed into *B. subtilis* PY79. For selection of correctly integrated strains, appropriate antibiotics were used and candidates were verified using diagnostic genomic DNA PCR and Sanger sequencing of PCR products.

### Fluorescence spectroscopy

For measurement of fluorescence intensity from reporter fusion strains, fresh cultured cells were used to inoculate 5 ml of modified glucose minimal media. That was cultured overnight while shaking at 37 °C. An aliquot was then diluted 1:100 in 100 ml glucose minimal media supplemented as necessary with antibiotics. These cells were incubated shaking at 37 °C until reaching an OD_600_∼0.3, whereupon they were pelleted after exposure of different concentration of TPEN from 10 to 100 μM, washed with two volume of 1 × PBS buffer (pH 7.4) before fixation. And then, the cell pellet was soaking in the 4% paraformaldehyde buffer for 20 min at room temperature^42^. After that, fixed cells were washed twice and resuspended in 5 ml 1 × PBS buffer (pH 7.4). OD_600_ readings were taken of the resuspended cells, and each cell sample was diluted to an OD_600_ of 0.2 with 1 × PBS buffer (pH 7.4). Fluorescence intensity of each 1 ml sample was taken with a Perkin-Elmer LS55 luminescence spectrometer.

### Western blot analysis of BsZur-FLAG

Cells were prepared under the same condition, as described in Fig. 2 for Supplementary Fig. 3d or Fig. 3b for Supplementary Fig. 3e. The cell pellet was resuspended in 0.5 ml of PBS buffer and lysed by sonication. Cell debris were removed by centrifugation and the resulting supernatant was mixed with SDS–PAGE loading buffer, and boiled at 95 °C for 10 min before being resolved by 13% SDS–PAGE. The proteins were transferred to a membrane at 60 mA for 40 min. The membrane was then blocked with blocking solution (Dry Milk dissolved in 20 mM Tris–HCl (pH 7.8), 150 mM NaCl and 0.1% Triton X-100) overnight. The membrane was incubated with a 1:1,000 dilution of polyclonal anti-FLAG Antibody (Sigma Chemical Co., SAB4301135)f or 1 h, washed with TBST (20 mM Tris, 150 mM NaCl and 0.1% Triton X-100), and incubated with 1:5,000 dilution of anti-rabbit IgG secondary antibody conjugated with alkaline phosphatase antibody (Santa Cruz Biotech, SC-2004) for 1 h. The membrane was then developed with 5 ml AP buffer (100 mM Tris–HCl (pH 9.5), 100 mM NaCl and 5 mM MgCl_2_), 1:100 dilution of NBT, and BCIP. The Zur-FLAG protein has a molecular weight of ∼17 kDa, in agreement with the observed mobility.

### Data availability

The authors declare that the data supporting the findings of this study are available within the article and its Supplementary Information files, or from the corresponding author on request.

## Additional information

**How to cite this article:** Shin, J.-H. & Helmann, J. D. Molecular logic of the Zur-regulated zinc deprivation response in *Bacillus subtilis*. *Nat. Commun.* 7:12612 doi: 10.1038/ncomms12612 (2016).

## Supplementary Material

Supplementary Information Supplementary Figures 1-9 and Supplementary Tables 1-2

## Figures and Tables

**Table 1:** DNA-binding affinity of wild-type and mutant Zur proteins on target promoters.

The Zur–DNA-binding affinity *K*_d_ (nM)*			
Promoters	WT	C84S (site II)†	H124A (site III)‡
*zinT*	15.6±3.5	75.5±5.3	96.0±1.3
*rpmEB*	14.3±2.6	77.9±4.0	101±2.3
*yciC*	25.4±1.1	80.6±3.2	129.0±3.8
*znuA*	12.5±4.0	73.0±2.0	110.2±3.0
*folEB*	21.9±3.9	28.6±2.5	100±4.0
*rpsNB*	14.3±2.5	16.5±2.5	99.0±1.6

^*^Average *K*_d_ values (±s.d., *n*=3) against *Bs*Zur monomer. Shaded cells correspond to high-affinity interactions that correlate to *in vivo* repression. Each *K*_d_ value was calculated by fitting data (representative data are shown in Supplementary Fig. 6) to a Hill equation, using SigmaPlot 2001 software (SPSS Inc).

^†^Regulatory Zn^2+^-binding site in *Bs*Zur.

^‡^Affects dimerization, this is a conserved metal binding site in some Fur family proteins.
