# Supplementary material for: Molecular logic of the Zur-regulated zinc deprivation response in Bacillus subtilis
Source: Nat Commun. 2016 Aug 26;7:12612. doi: 10.1038/ncomms12612 (PMC5007448; doi:10.1038/ncomms12612)
Supplement: Supplementary Information — Supplementary Figures 1-9 and Supplementary Tables 1-2 [file ncomms12612-s1.pdf]

Supplementary Information

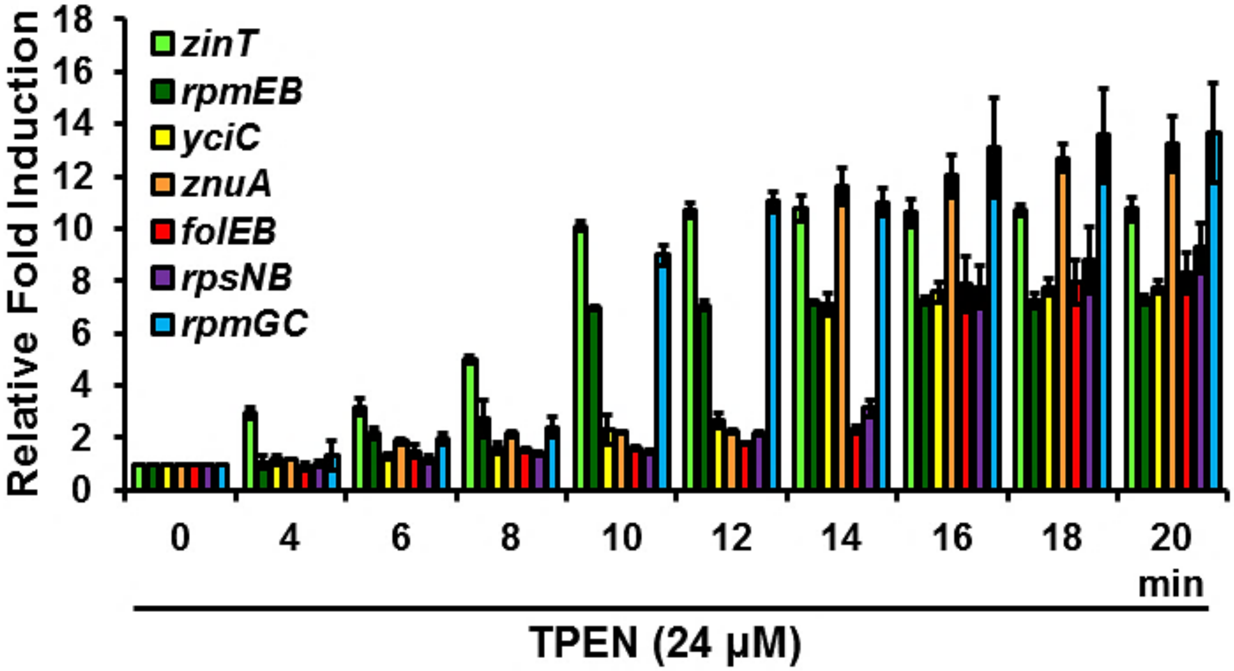

Supplementary Figure 1 | Time-dependent induction of Zur target genes in response to the zinc specific chelator TPEN. Wild-type cells were grown in liquid LB to early exponential phase and treated with 24 μM TPEN for periods of time from 4 to 20 minutes. RNA samples were analyzed by S1 nuclease mapping. Relative fold induction is presented by using average values from three independent experiments with the value of the untreated sample set as 1.

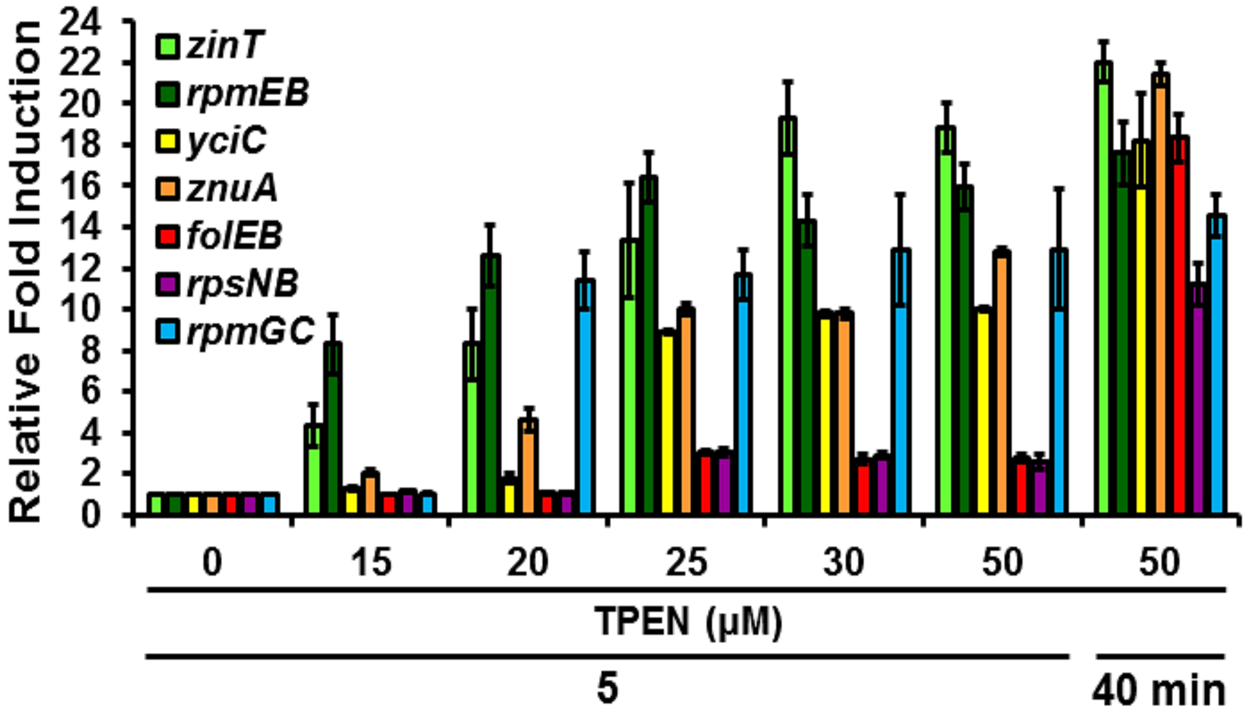

Supplementary Figure 2 | Concentration- and time-dependent induction of Zur target genes in response to TPEN. WT cells were grown in LB medium to early exponential phase and treated with various concentrations of TPEN (from 15 to 50 µM; final) for 5 minutes, or for 40 minutes at 50 µM to allow full derepression of all Zur target genes. RNA samples were analyzed by S1 nuclease mapping. Average relative fold induction from three independent experiments is presented as the normalized values to the untreated sample set as 1.

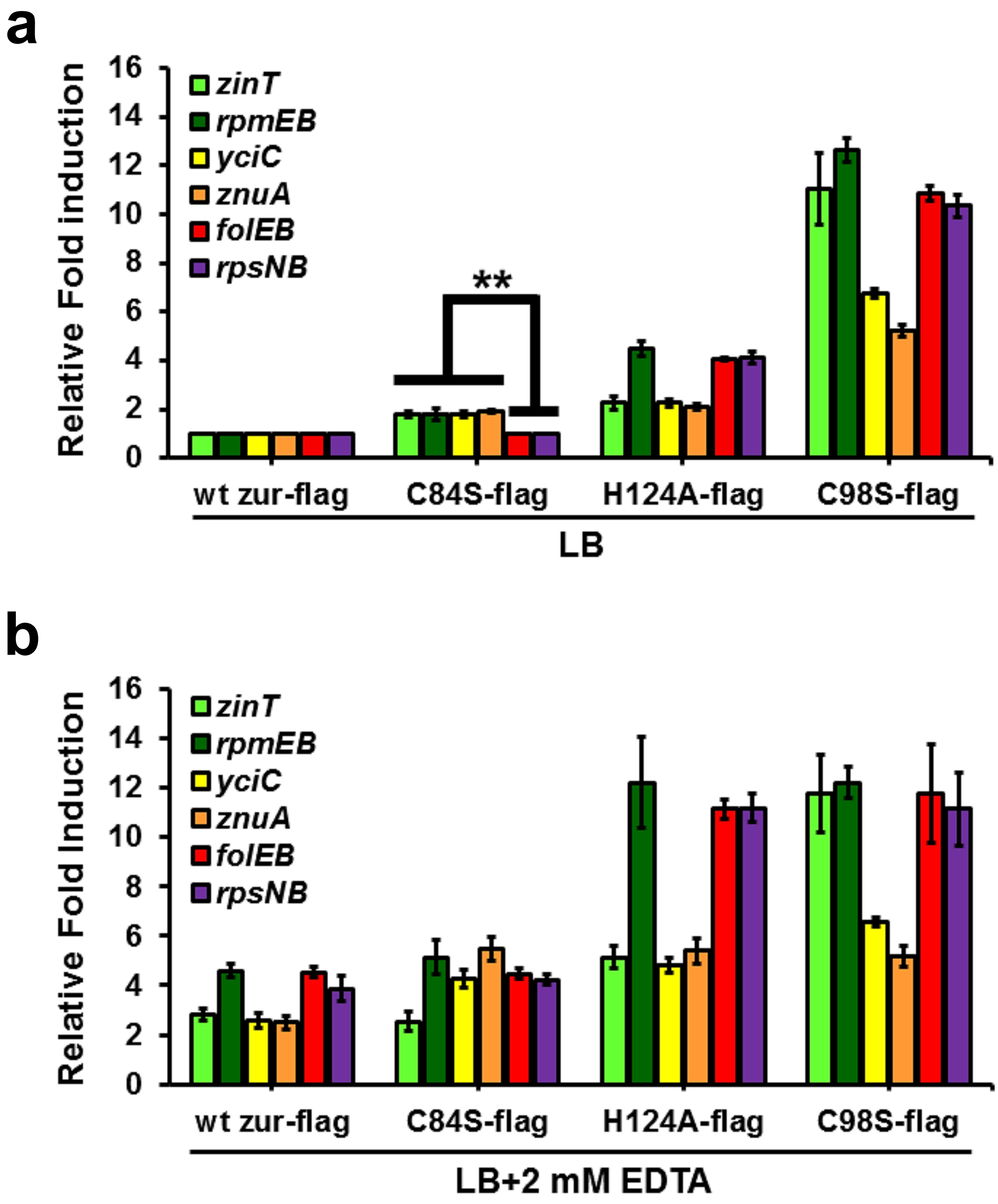
a
Relative Fold induction
16
14
12
10
8
6
4
2
0
zinT
rpmEB
yciC
znuA
folEB
rpsNB
**
wt zur-flag
C84S-flag
H124A-flag
C98S-flag
LB
b
Relative Fold Induction
16
14
12
10
8
6
4
2
0
zinT
rpmEB
yciC
znuA
folEB
rpsNB
wt zur-flag
C84S-flag
H124A-flag
C98S-flag
LB+2 mM EDTA

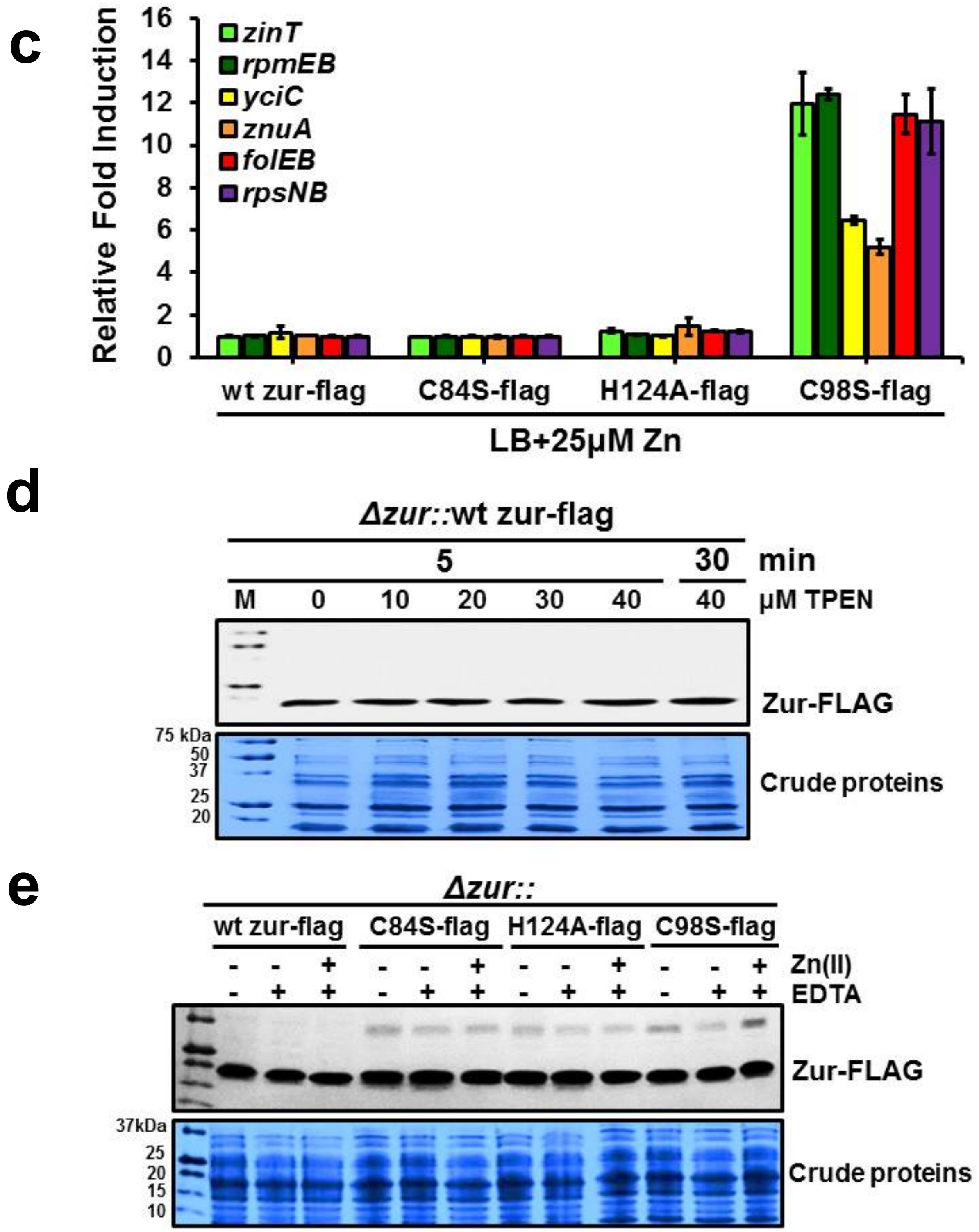
c
zinT
rpmEB
yciC
znuA
folEB
rpsNB
Relative Fold Induction
0
2
4
6
8
10
12
14
16
wt zur-flag
C84S-flag
H124A-flag
C98S-flag
LB+25μM Zn
d
Δzur::wt zur-flag
5
30
min
M
0
10
20
30
40
40
μM TPEN
Zur-FLAG
75 kDa
50
37
25
20
Crude proteins
e
Δzur::
wt zur-flag
C84S-flag
H124A-flag
C98S-flag
Zn(II)
EDTA
Zur-FLAG
37kDa
25
20
15
10
Crude proteins

Supplementary Figure 3 | Quantitative analysis of the reversible transcriptional repressor activity of FLAG-tagged WT and mutant Zur proteins. Average amounts of transcripts in Figure 3b are presented with normalized values from three independent experiments (**a**) in LB medium, (**b**) in LB + 2 mM EDTA, and (**c**) in LB+25 μM $ZnSO_4$. Steady-state levels of FLAG-tagged Zur variant proteins in cells. (**d**) Effects of TPEN on Zur protein expression in WT. (**e**) Effects of Zn^{2+} and EDTA in the complemented strains with WT and mutant Zur proteins. Cells were prepared under the same conditions as described in Figure 2 for (**d**) and for Figure 3b for (**e**). 50 μg of crude proteins was loaded per lane. Crude extracts were used as a loading control. The levels of Zur protein were detected by immunoblotting using anti-FLAG antibodies as described in Methods.

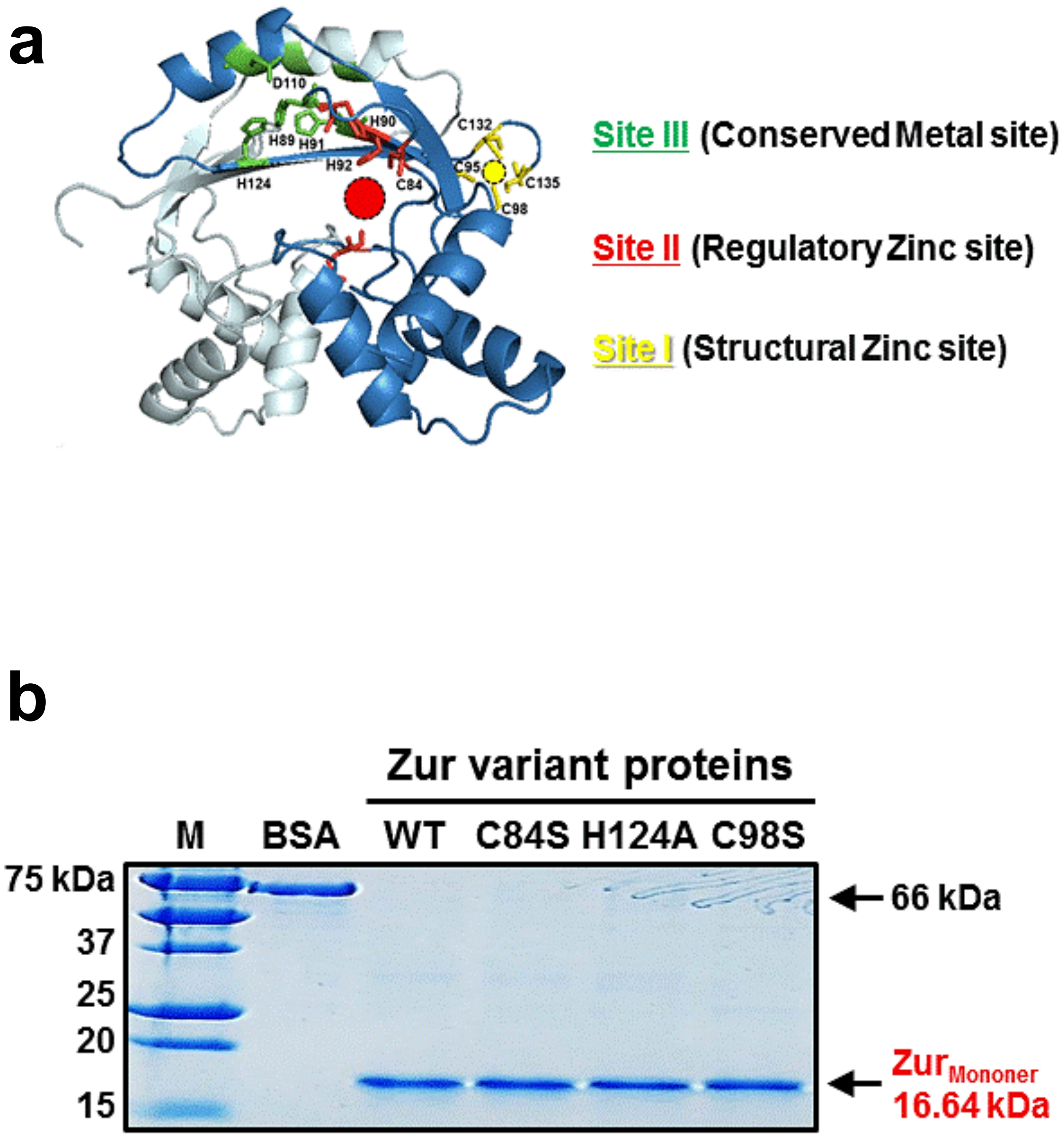
a
D110
H89
H91
H90
H92
C84
H124
C132
C95
C135
C98
Site III (Conserved Metal site)
Site II (Regulatory Zinc site)
Site I (Structural Zinc site)
b
Zur variant proteins
M
BSA
WT
C84S
H124A
C98S
75 kDa
37
25
20
15
66 kDa
Zur Mononer
16.64 kDa

c

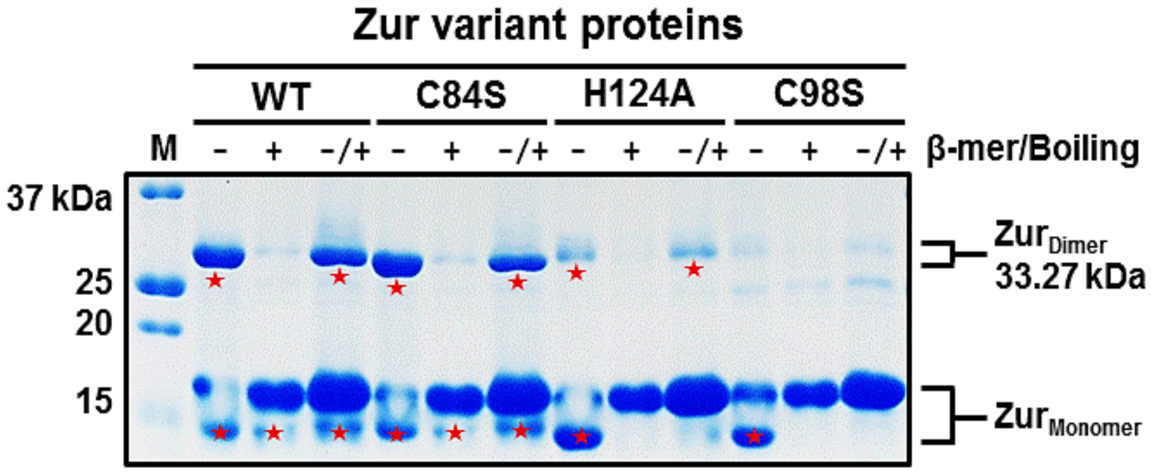

d

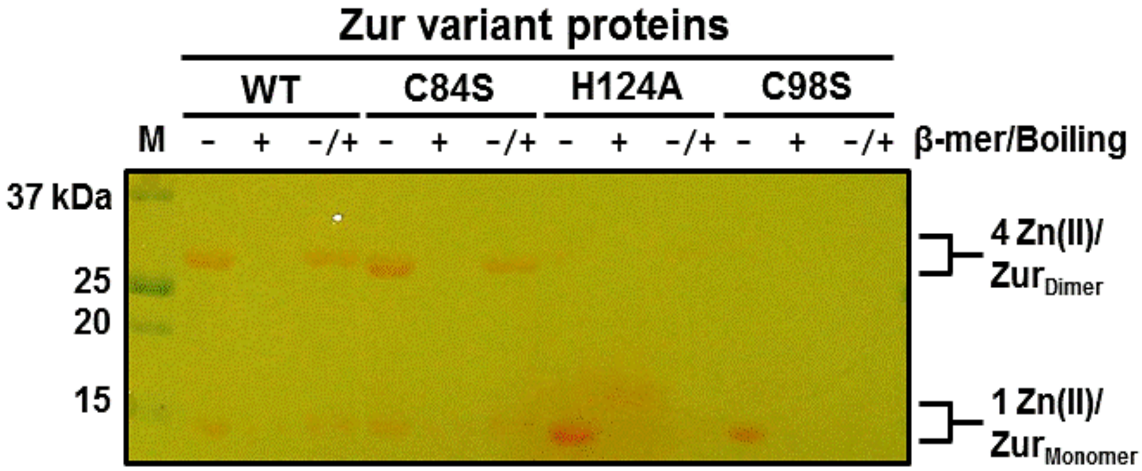

Supplementary Figure 4 | Modeled BsZur structure (against ScZur crystal structure; PDB:3MWM) and the effects of ligand substitution on dimerization and Zn(II) occupancy. (**a**) Two Zn(II) binding sites and one conserved putative metal binding site (no Zn(II) binding) with putative ligands (as sticks) from site I (yellow colored circle), site II (red closed circle) and the putative site III (green) are highlighted in the BsZur dimer model. (**b**) SDS-PAGE gel to demonstrate purity of the purified Zur WT and mutant proteins. (**c**) Coomassie stained PAGE gel for detection of BsuZur oligomeric status. WT and mutant Zur proteins (7 µg) were subjected to 13% SDS-PAGE. To visualize dimeric forms better, boiled samples in SDS-loading dye were mixed with non-boiled protein before loading. C98S, C84S, and H124A variants represent mutations in the site I, II, and conserved metal site (site III), respectively. Red colored-star (★) indicates Zn(II)-containing protein bands. (**d**) PAR stained of non-reducing SDS-PAGE gel image for detection of Zn(II) in proteins.

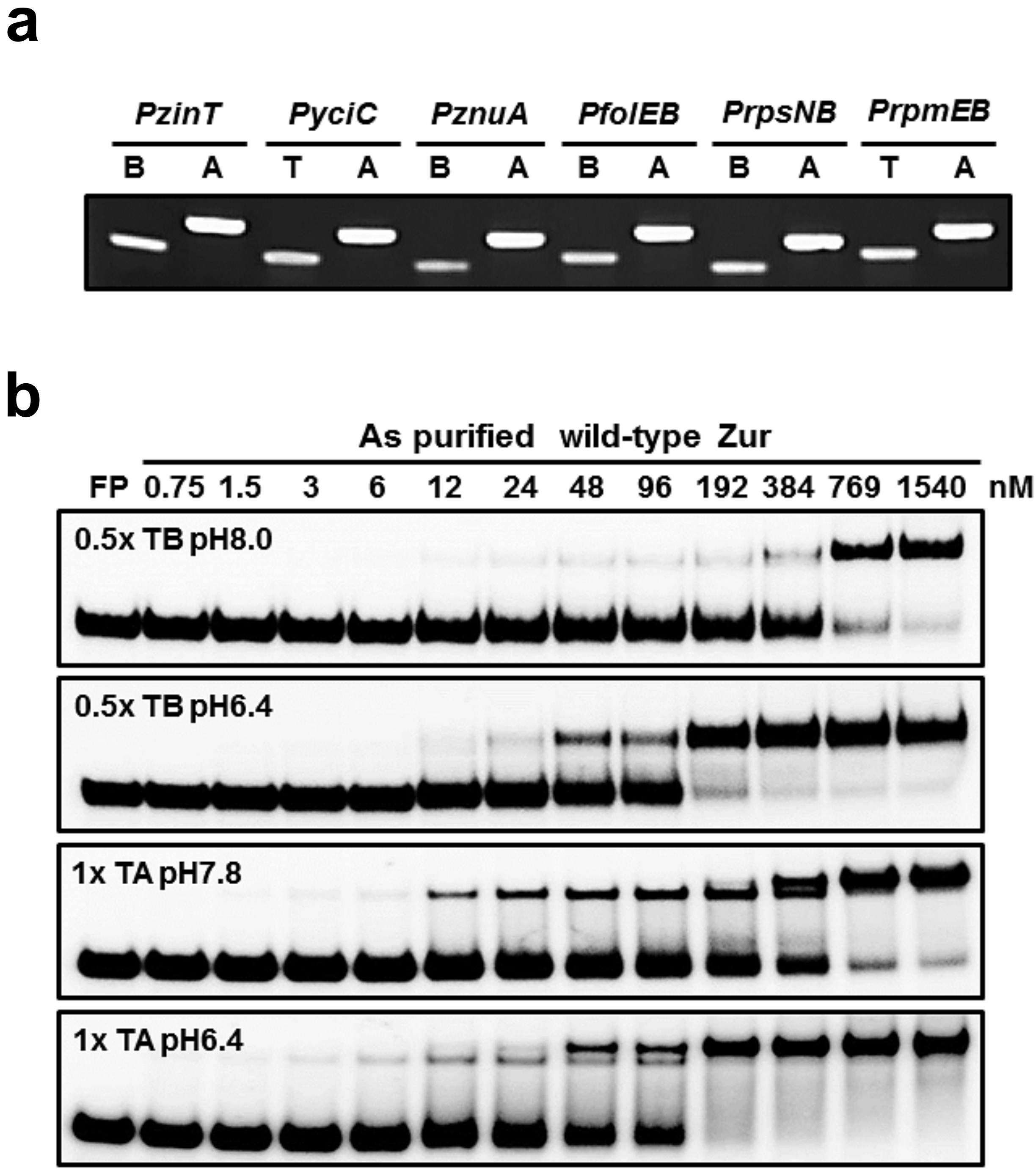

Supplementary Figure 5 | Purification of Zur target promoter DNAs and determination of optimal DNA-binding conditions. (**a**) EtBr stained gel image. B, T, and A indicate bottom, top primer, and annealed double strand DNAs, respectively, for reach target operator. 100 pmole of primer or 100 ng of eluted DNAs were loaded onto an 8% polyacrylamide gel. (**b**) EMSA was carried out in a variety of different binding conditions to optimize Zur binding on the *zinT* promoter DNA at the presence of 0.1 μM $ZnSO_4$.

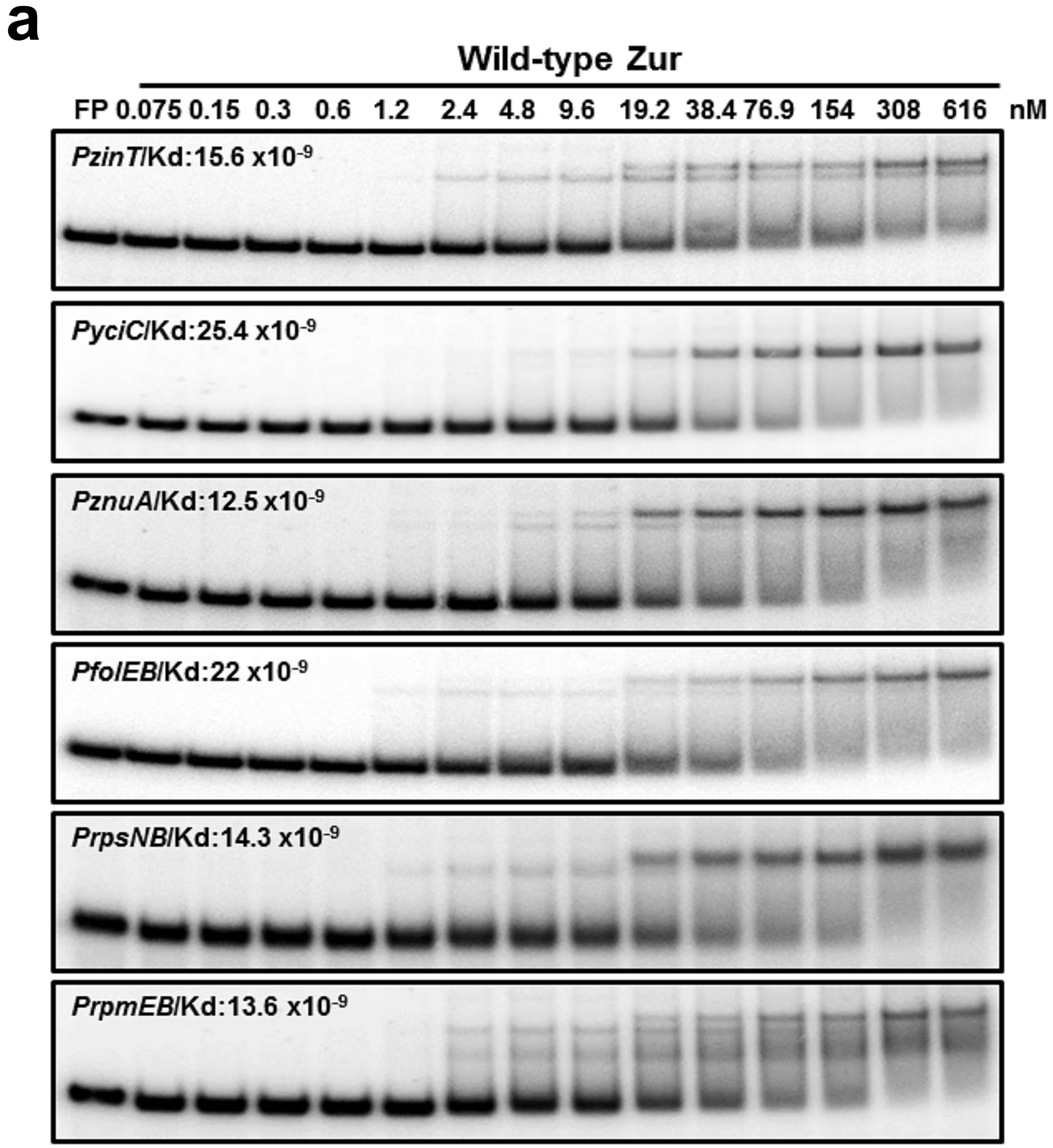
a
Wild-type Zur
FP 0.075 0.15 0.3 0.6 1.2 2.4 4.8 9.6 19.2 38.4 76.9 154 308 616 nM
PzinT/Kd:15.6 x10-9
PyciC/Kd:25.4 x10-9
PznuA/Kd:12.5 x10-9
PfolEB/Kd:22 x10-9
PrpsNB/Kd:14.3 x10-9
PrpmEB/Kd:13.6 x10-9

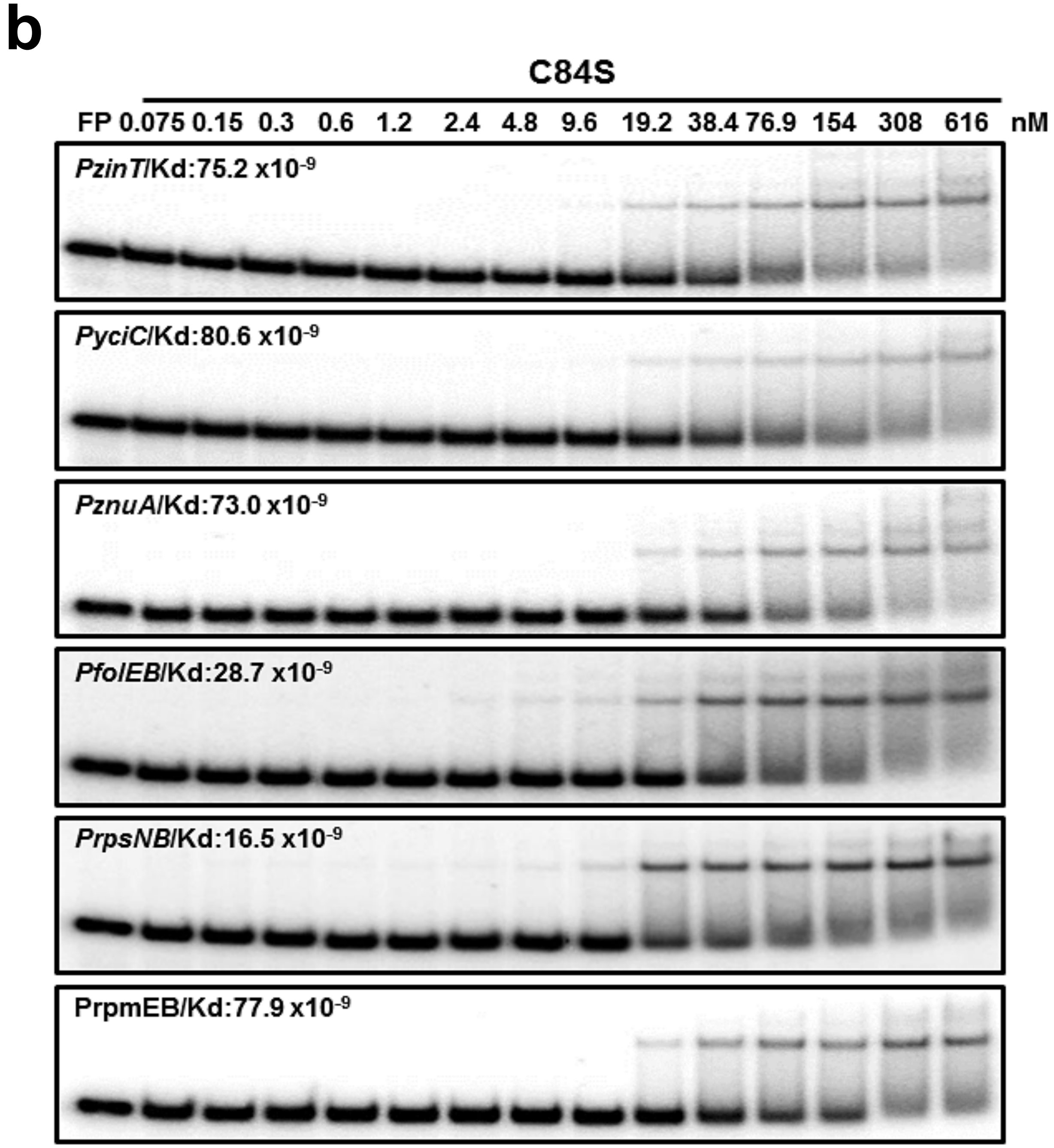
b
C84S
FP 0.075 0.15 0.3 0.6 1.2 2.4 4.8 9.6 19.2 38.4 76.9 154 308 616 nM
PzinT/Kd:75.2 x10-9
PyciC/Kd:80.6 x10-9
PznuA/Kd:73.0 x10-9
PfolEB/Kd:28.7 x10-9
PrpsNB/Kd:16.5 x10-9
PrpmEB/Kd:77.9 x10-9

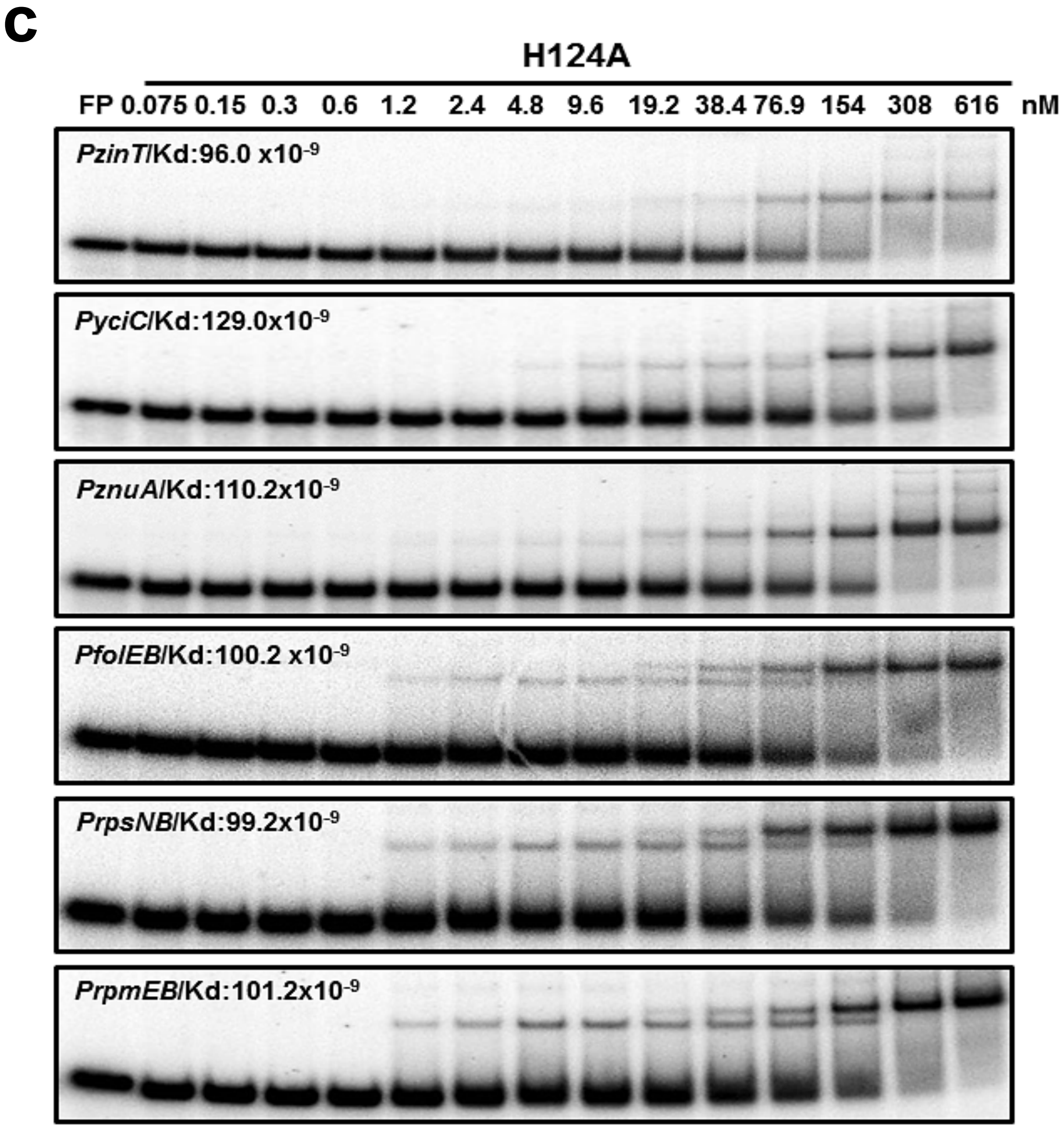

Supplementary Figure 6 | Zur-DNA binding affinitied determined by EMSA for different Zur variants and promoters. Each labeled promoter probe of 45 bp (*PzinT*, *PyciC*, *PznuA*, *PfolEB*, *PrpsNB*, and *PrpmEB*) was incubated with increasing amounts of purified WT (**a**), C84S (**b**), and H124A (**c**) Zur proteins. The band intensity of unbound DNA was measured against Zur concentration by using Multi Gauge V3.0 software. Each K_d value was calculated by fitting data to a Hill equation using Sigma Plot 2001 (SPSS Inc) from three independent experiments and is presented at the top from left on the gel image, respectively.

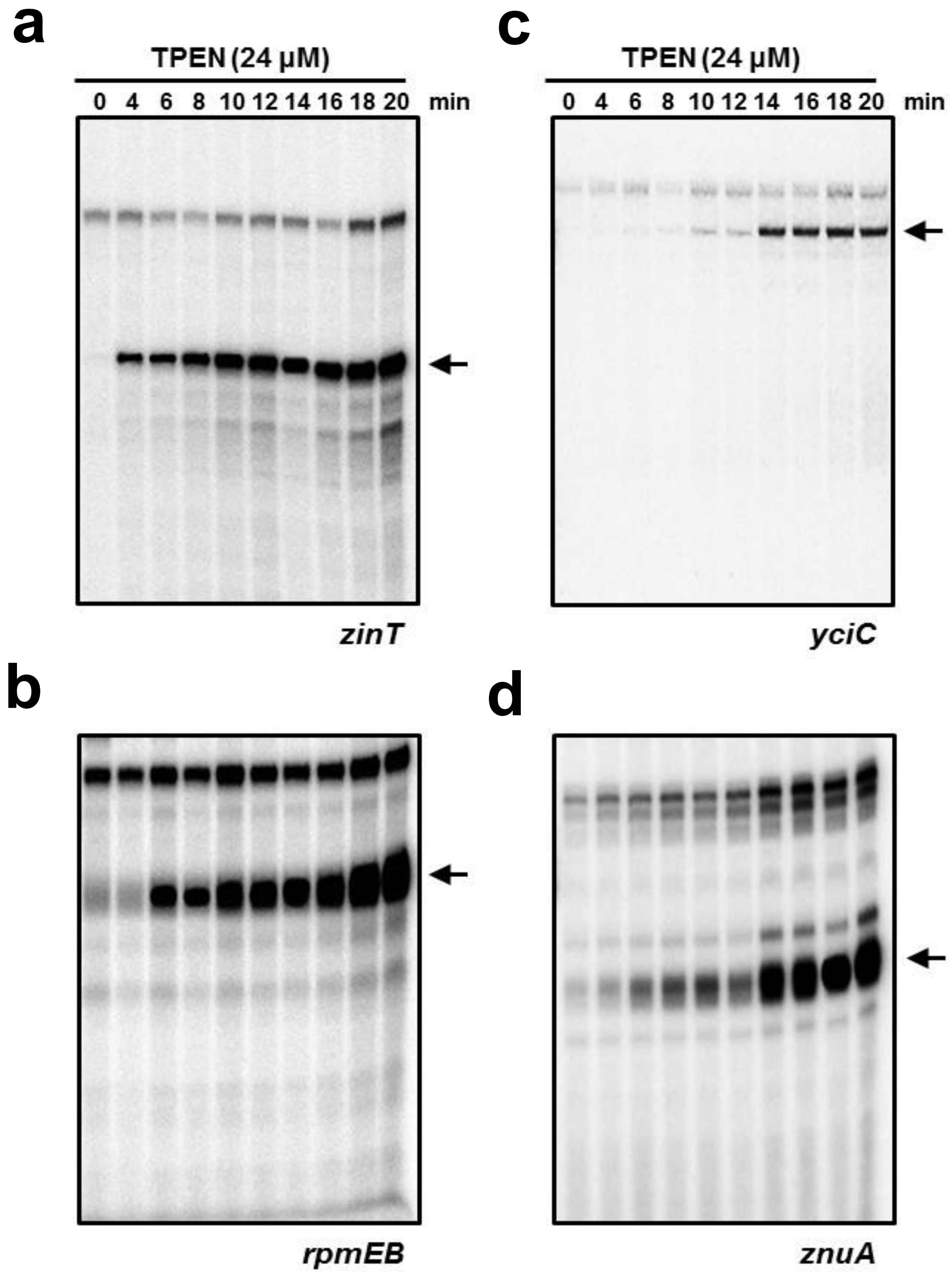
a
TPEN (24 μM)
0 4 6 8 10 12 14 16 18 20 min
zinT
b
rpmEB
c
TPEN (24 μM)
0 4 6 8 10 12 14 16 18 20 min
yciC
d
znuA

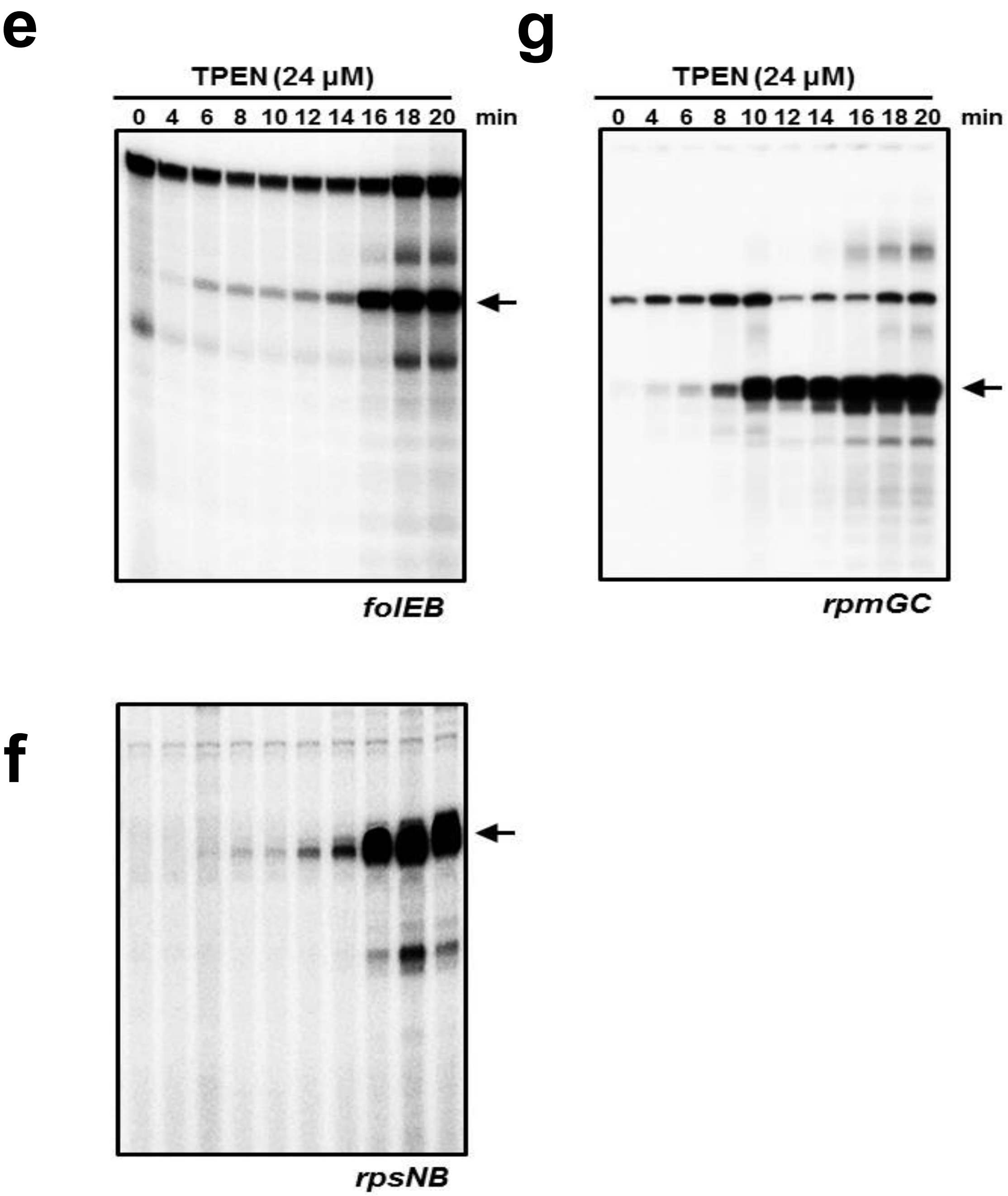

Supplementary Figure 7 | S1 nuclease protection analysis of Zur traget genes in wild type strain. RNA samples taken at 2 min intervals were analysed over a 20 min time course after treatment with 24 µM TPEN (see Figure 1a for details). Arrows indicate the Zur target promoter for each gene. (**a**) *zinT*, (**b**) *rpmEB*, (**c**) *yciC*, (**d**) *znuA*, (**e**) *folEB*, (**f**) *rpsNB*, (**g**) *rpmGC*. For each sample, Average values from three independent experiments are presented in Supplementary Figure 1.

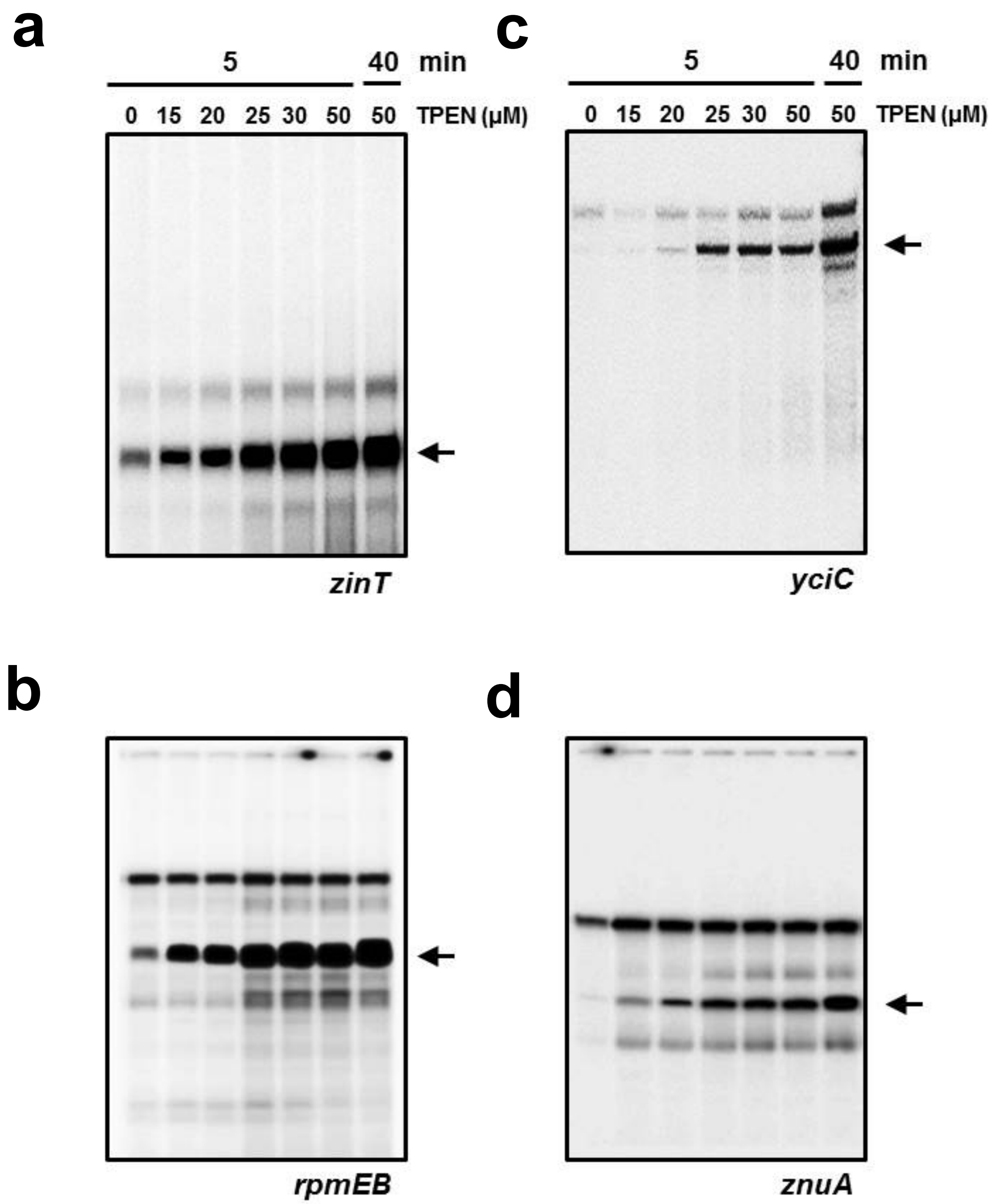
a
5
40
min
0
15
20
25
30
50
50
TPEN (μM)
zinT
b
rpmEB
c
5
40
min
0
15
20
25
30
50
50
TPEN (μM)
yciC
d
znuA

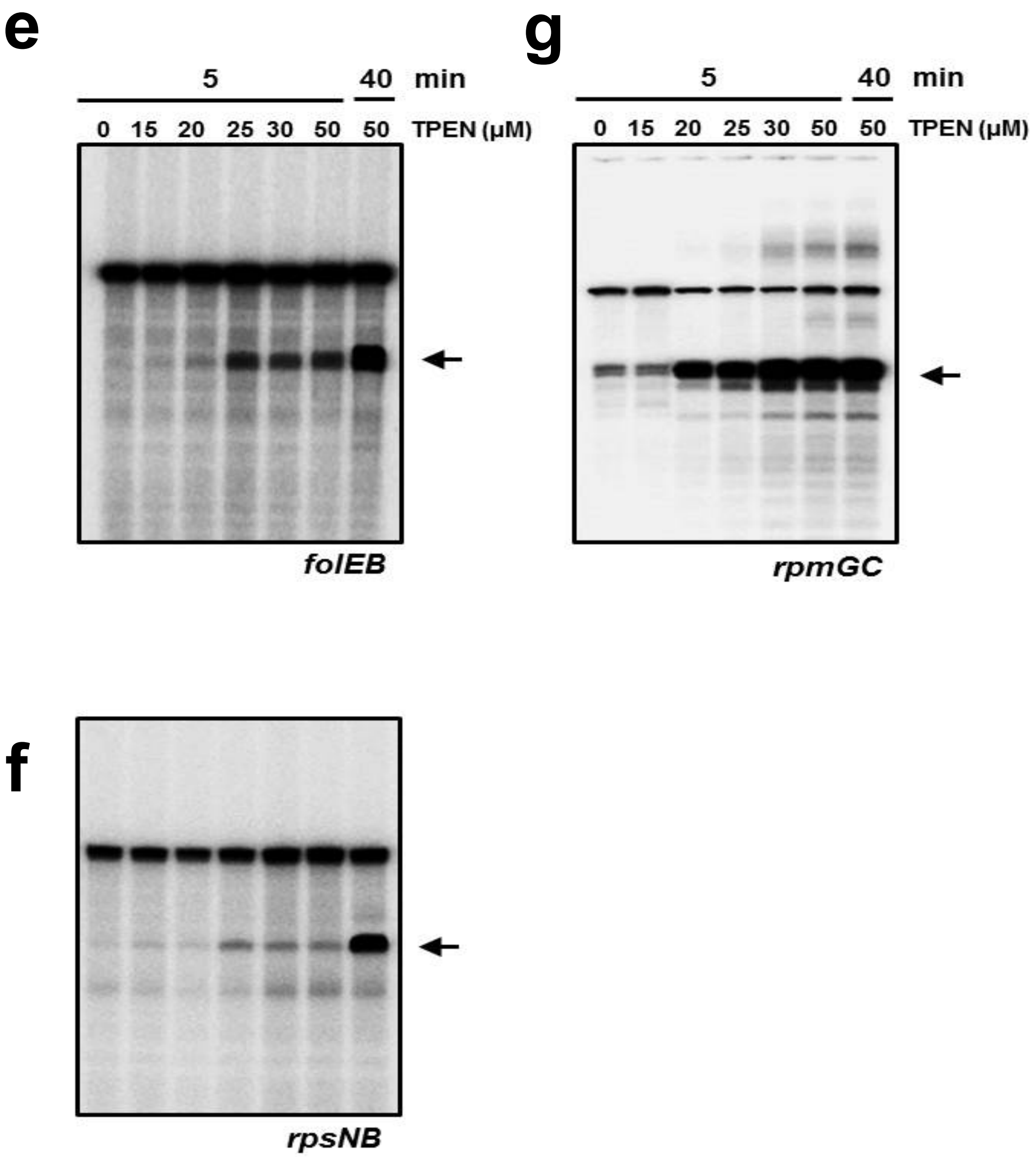

Supplementary Figure 8 | S1 nuclease mapping of Zur traget genes. Wild-type cells were grown to early exponential phase (OD_{600} ~0.5) in liquid LB medium and treated with the zinc chelator TPEN. Cells were treated with variable levels of TPEN (15 to 50 µM) for 5 minutes or with 50 µM for 40 minutes to fully induce all Zur target genes (**a**) *zinT*, (**b**) *rpmEB*, (**c**) *yciC*, (**d**) *znuA*, (**e**) *folEB*, (**f**) *rpsNB*, (**g**) *rpmGC*. (see Figure 1b for details). Arrows indicate the Zur target promoter for each gene. For each sample, Average values from three independent experiments are presented in Supplementary Figure 2.

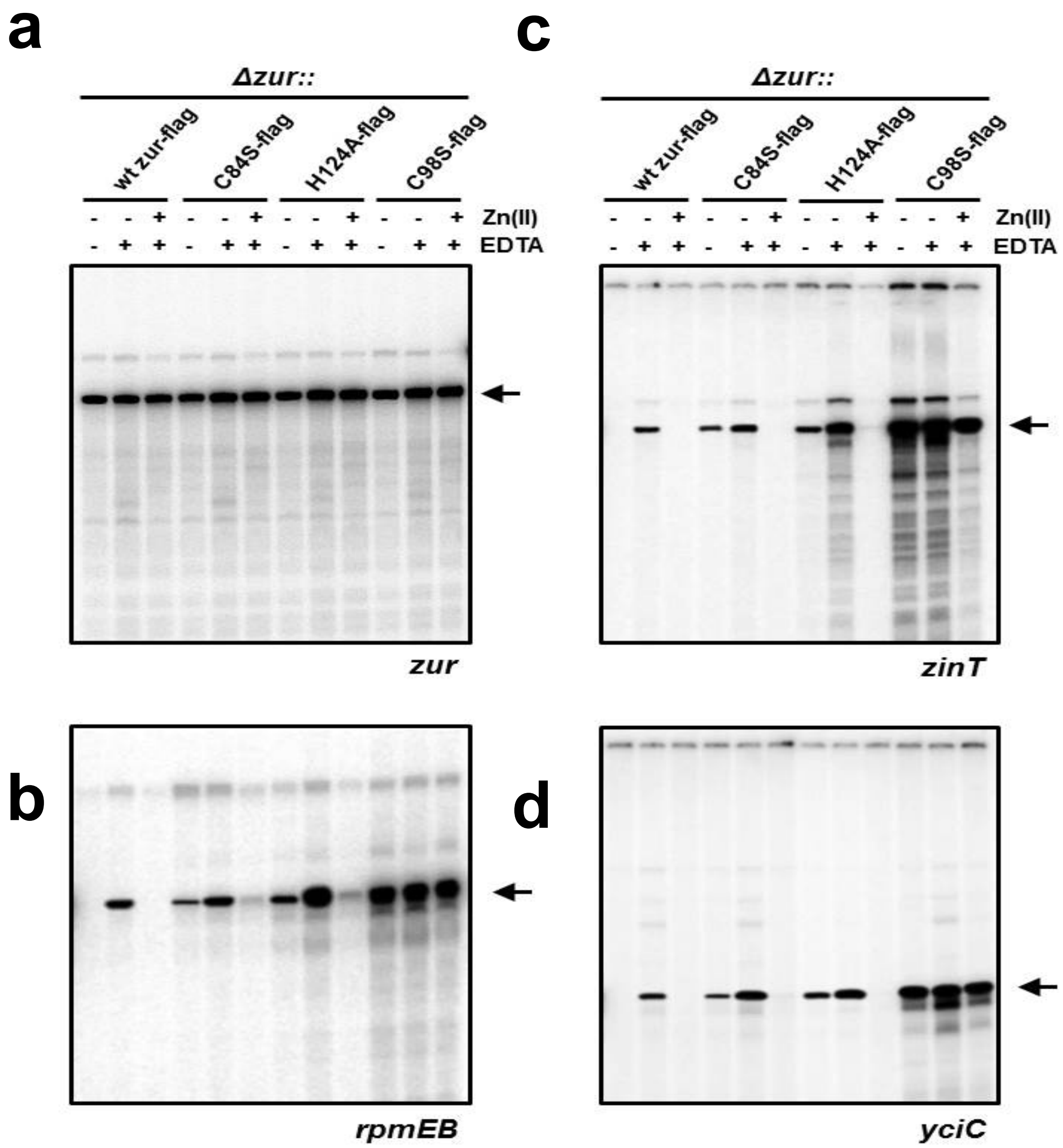

a
b
c
d
Δzur::
wt zur-flag
C84S-flag
H124A-flag
C98S-flag
Zn(II)
EDTA
zur
rpmEB
zinT
yciC

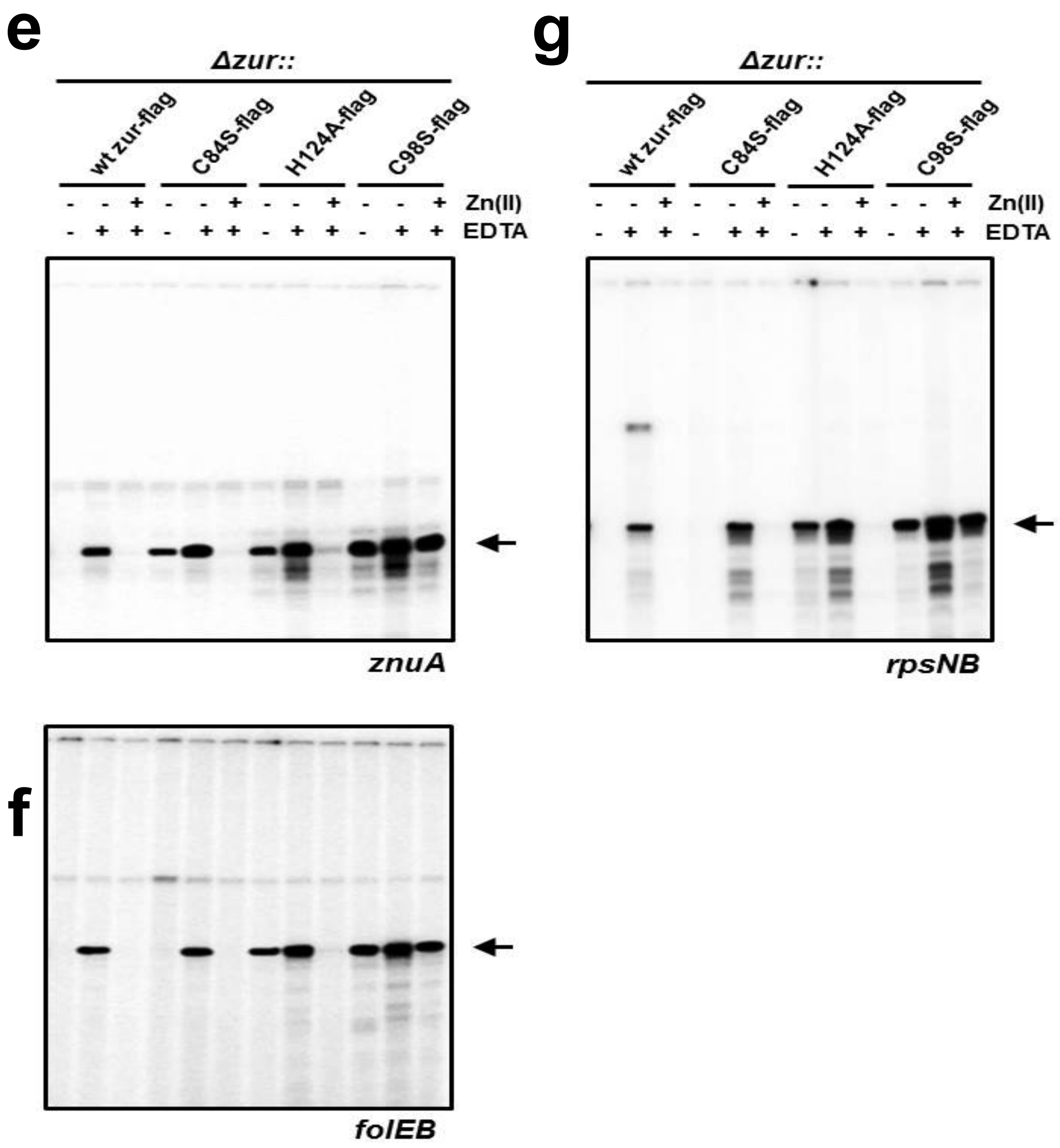

Supplementary Figure 9 | S1 nuclease protection analysis of Zur traget genes for reversible transcriptional repressor. Expression profiles of six Zur target genes in complemented strains with FLAG-tagged WT or mutant Zur proteins under the same conditions as (Figure 3**b**). Wild-type and complemented strains with FLAG-tagged Zur variant proteins were grown to early exponential phase (OD600 ~0.5) in liquid LB medium and treated with metal chelating EDTA (2 mM) for full derepression of all Zur target genes and then added 25 μM ZnSO4 for re-repression by Zur variant proteins. Average values from three independent experiments are presented in Supplementary Figure 3a, b, and c. Transcripts of the *zur* gene and ribosomal RNA were used as constitutive expression controls.

Supplementary Tables

Supplementary Table 1 Strains and plasmids used in this study

**Strain /plasmid**		**Genotype / description**	**Source**
***B. subtilis* strains**			
Cu1065	HB17502	Wild-type strain (*trpC2* /attSPβ)	Lab stock
	HB17506	*Δzur::tet*^{R}	Lab stock
	HB17507	*Δzur::tet +amyE::*WT Zur-FLAG/*spec*^{R}	Lab stock
	HB17508	*Δzur::tet +amyE::*C98S Zur-FLAG/*spec*^{R}	Lab stock
	HB17509	*Δzur::tet +amyE::*H124A Zur-FLAG/*spec*^{R}	Lab stock
	HB17510	*Δzur::tet +amyE:* C84S Zur-FLAG/*spec*^{R}	Lab stock
PY79	HB17563	Prototrophic derivative of *B. subtilis* 168	Lab stock
	HB17657	*thrC::egfp(chl*^{R}*/kan*^{R}*)+amyE::ecfp*(*chl*^{R})+lacA::mCherry(*spec*^{R})	This study
	HB17659	*thrC::PrpmEB-egfp*(*chl*^{R}*/kan*^{R})+ *amyE::PznuA-ecfp*(*chl*^{R})+*lacA::PrpsNB-mCherry*(*spec*^{R})	This study
	HB17661	*thrC:: PznuA-egfp*(*chl*^{R}*/kan*^{R})+ *amyE:: PrpsNB-ecfp*(*chl*^{R})+*lacA:: PrpmEB-mCherry*(*spec*^{R})	This study
***E. coli* strains**			
DH5α	HE17502	F– Φ80*lac*ZΔM15 Δ(*lac*ZYA-*arg*F) U169 *rec*A1 *end*A1 *hsd*R17 (rK–, mK+) *pho*A *sup*E44 λ– *thi*-1 *gyr*A96 *rel*A1	Lab stock
	HE17513	pET3a::BsuZurWT	This study
	HE17514	pET3a::BsuZurC84S	This study
	HE17515	pET3a::BsuZurC98S	This study
	HE17516	pET3a::BsuZurH124A	This study
BL21	HE17501	(DE3/pLysS)	Lab stock
	HE17517	pET3a	This study
	HE17518	pET3a::BsuZurWT	This study
	HE17519	pET3a::BsuZurC84S	This study
	HE17520	pET3a::BsuZurC98S	This study
	HE17521	pET3a::BsuZurH124A	This study

**Plasmids**		
pET3a	Over expression vector in *E.coli*	Novagen
pEGFP_Star *thrC*	Integration vector, chl^R/kan^R	This study
pECFP_Star *amyE*	Integration vector, chl^R	This study
pmCherry_Star *lacA*	Integration vector, $spec^R$	This study

Supplementary Table 2 Oligonucleotides used in this study

Assays and primers	Primer sequence (5' to 3')	Description
**EMSA***		
JHS008	ATGTATGTATAA**TAAATCGTAATGTTTTCGATTTA**ATAAAAAAGG	*zinT* Top
JHS009	CCTTTTTTAT**TAAATCGAAAACATTACGATTTA**TTATACATACAT	*zinT* Bottom
JHS010	ACCATACAATT**TAAGTCGTAACAATTACGTTTTA**TGATAAATGGC	*yciC* Top
JHS011	GCCATTTATCA**TAAAACGTAATTGTTACGACTTA**AATTGTATGGT	*yciC* Bottom
JHS014	ATTTATTCTTG**CAAAACGTAATGACTTCGGTTTA**TTATGATATAG	*znuA* Top
JHS015	CTATATCATAA**TAAACCGAAGTCATTACGTTTTG**CAAGAATAAAT	*znuA* Bottom
JHS018	ACCGATCAAAA**TAAATAGTAATTATTACGATTTG**TTAGTGAGAGG	*folEB* Top
JHS019	CCTCTCACTAA**CAAATCGTAATAATTACTATTTA**TTTTGATCGGT	*folEB* Bottom
JHS020	GAATGATACATTT**TAAATCGTAACAATTTCGATTTA**AAGGGAGGC	*rpsNB* Top
JHS021	GCCTCCCTT**TAAATCGAAATTGTTACGATTTA**AAATGTATCATTC	*rpsNB* Bottom
JHS022	GTAAGGTTATAAAA**TAAAACGTAATTATTACGATTTA**TTGAAAGG	*rpmEB* Top
JHS023	CCTTTCAA**TAAATCGTAATAATTACGTTTTA**TTTTATAACCTTAC	*rpmEB* Bottom
**S1 mapping analysis**		
JHS024	GGTGCTGTCACATTGAAGCGG	*zinT* S1 F
JHS025	GACTCACCTGCAGAAGAACCGG	*zinT* S1 R
JHS026	CCCTGAACATACAAAAGTGGTG	*yciC* S1 F
JHS027	GATAACCGCTCAGTACGGTAACCGG	*yciC* S1 R
JHS028	CTAATGATTGAAGTAGCCCGGC	*znuA* S1 F
JHS029	CACCCTTTGAATCCGCCGACCC	*znuA* S1 R
JHS030	GACGGACACGCGTTTGTGCGTG	*folEB* S1 F
JHS031	CTCTTTTTCCACTGGTTTCTCGCC	*folEB* S1 R
JHS032	GAATGGGCGGCTTCAGGCCGTC	*rpsNB* S1 F
JHS033	CCTTCAACTCGCGTCTAATACCAGC	*rpsNB* S1 R
JHS034	CGATGTGAAACAGAGGGAAGG	*rpmEB* S1 F
JHS035	CGGTAGCCGCTGTTGACATCCTG	*rpmEB* S1 R
JHS097	GCATATGATTTCTTAGAGGTAAACGTCC	*rpmGC* S1 F
JHS098	CAGCTCCAAACGGTCTGGATTGGTGCG	*rpmGC* S1 R
**Quantitative PCR**		
JHS221	GGCCATCCGCATGAAGAGCATTTTTCCTT	*zinT* qPCR F
JHS222	AAGGAAAAATGCTCTTCATGCGGATGGCC	*zinT* qPCR R
JHS224	GGATGTATCAGGCTGACCATTCCAGATGCGA	*yciC* qPCR F
JHS225	TGCCTTTCCGCTTTTCACAGATGCACAGA	*yciC* qPCR R
JHS206	GAGCGGTTTGTTTGTGATTG	*znuA* qPCR F

	JHS207	ACGTGCAGTTTATCACCCTTT	*znuA* qPCR R
	JHS218	TTCAACAGTGAACCGATCAAA	*folEB* qPCR F
	JHS219	AAGTATTGAAGACGCTCCGTT	*folEB* qPCR R
	JHS234	GGGCGGCTTCAGGCCGTCTGTTT	*rpsNB* qPCR F
	JHS235	CCAGCGTATTGCTCAACAAGCTGTTGGCGT	*rpsNB* qPCR R
	JHS236	CCATGAGGTTTACGATGTGAAACAGAGGGAAGGA	*rpmEB* qPCR F
	JHS237	AAACGGTAGCCGCTGTTGACATCCTGA	*rpmEB* qPCR R
**Fluorescence fusion plasmid**			
	JHS256	GAGGGGTGCTGTCACACCCCCGGGGGTCGATATGG	*PzinT Xma*I F
	JHS257	GAAAATAAAATGTTCATTCCAACCCCCGGGGGTTATTAAATCG	*PzinT Xma*I R
	JHS258	CTGAACATACCCCCGGGGGGATCAGCTGAATTGAAAACCG	*PyciC Xma*I F
	JHS261	GGAATTTTTTTCCCCCCGGGGGCTCCATTAAAATAGAATGATTACG	*PyciC Xma*I R
	JHS262	ATGATTGAAGTCCCCCGGGGGTTTAGCCGGGTTTTTTTAGTAC	*PznuA Xma*I F
	JHS263	CATATCGTATACCCCCGGGGGTTCAGTAACTTTAAATCAAAATG	*PznuA Xma*I R
	JHS264	CGCGGAAAGCCCCCCGGGGGATGTTTAGGATTTTGACGGACAC	*PfolEB Xma*I F
	JHS265	CATTCAGAAAACCCCCGGGGGTAACAAATCGTAATAATTAATATT	*PfolEB Xma*I R
	JHS266	GAGACAATGCCCCCCGGGGGACATAAAAAAGAGCTCATTAAGC	*PrpsNB Xma*I F
	JHS267	TTTAGCCAAGTCCCCCGGGGGCCCTTTAAATCGAAATTGTTCG	*PrpsNB Xma*I R
	JHS268	TTATTTGCCCCCGGGGGTAGATTATTCTTCCTATTCTTAAATGC	*PrpmEB Xma*I F
	JHS269	CTTCTTTCCCCCGGGGGTCCTTTCAATAAATCGTAATAATTACG	*PrpmEB Xma*I R

*Zur binding sequences on the promoter region of each Zur target gene were presented as **bold** with underline.
